# Meta-Analyses of Splicing and Expression Quantitative Trait Loci Identified Susceptibility Genes of Glioma

**DOI:** 10.3389/fgene.2021.609657

**Published:** 2021-04-15

**Authors:** C. Pawan K. Patro, Darryl Nousome, The Glioma International Case Control Study (GICC), Elizabeth B. Claus, Rose K. Lai

**Affiliations:** School of Public Health, Yale University, New Haven, CT 06510, United States and Department of Neurosurgery, Brigham and Women’s Hospital, Boston, MA 02115, United States; Department of Epidemiology and Biostatistics, School of Public Health, Georgia State University, Atlanta, GA 30303, United States; Duke Cancer Institute, Duke University Medical Center, Durham, NC 27710, United States and Cancer Control and Prevention Program, Department of Community and Family Medicine, Duke University Medical Center, Durham, NC 27710, United States; Department of Public Health Sciences, School of Medicine, University of Virginia, Charlottesville, VA 22903, United States; Department of Population and Quantitative Health Sciences and the Cleveland Center for Health Outcomes Research, Case Western Reserve University School of Medicine, Cleveland, OH 44106, United States; Department of Epidemiology and Biostatistics, Memorial Sloan Kettering Cancer Center, New York, NY 10017, United States; Department of Epidemiology and Biostatistics, Memorial Sloan Kettering Cancer Center, New York, NY 10017, United States; Danish Cancer Society Research Center, Survivorship, Danish Cancer Society, Copenhagen 2100, Denmark; 15Oncology Clinic, Finsen Centre, Rigshospitalet, University of Copenhagen, Copenhagen 2100, Denmark; Department of Laboratory Medicine and Pathology, Mayo Clinic Comprehensive Cancer Center, Mayo Clinic, Rochester, MN 55905, United States; Department of Radiation Sciences, Umeå University, Umeå 901 87, Sweden; Department of Neurological Surgery, School of Medicine, University of California, San Francisco, CA 94143, United States; Division of Molecular Pathology, The Institute of Cancer Research, London SW7 3RP, United Kingdom; Department of Epidemiology and Population Health, Stanford Cancer Institute, Stanford University, Stanford, CA 94305, United States; ^1^Department of Neurology and Preventive Medicine, Keck School of Medicine, University of Southern California, Los Angeles, Los Angeles, CA, United States; ^2^Center for Prostate Disease Research, Department of Surgery, Uniformed Services University of the Health Sciences, Rockville, MD, United States

**Keywords:** glioma, quantitative trait loci, eQTL, SQTL, summary data based mendelian randomization analyses, GWAS, meta-analysis

## Abstract

**Background:**

The functions of most glioma risk alleles are unknown. Very few studies had evaluated expression quantitative trait loci (eQTL), and insights of susceptibility genes were limited due to scarcity of available brain tissues. Moreover, no prior study had examined the effect of glioma risk alleles on alternative RNA splicing.

**Objective:**

This study explored splicing quantitative trait loci (sQTL) as molecular QTL and improved the power of QTL mapping through meta-analyses of both *cis* eQTL and sQTL.

**Methods:**

We first evaluated eQTLs and sQTLs of the CommonMind Consortium (CMC) and Genotype-Tissue Expression Project (GTEx) using genotyping, or whole-genome sequencing and RNA-seq data. Alternative splicing events were characterized using an annotation-free method that detected intron excision events. Then, we conducted meta-analyses by pooling the eQTL and sQTL results of CMC and GTEx using the inverse variance-weighted model. Afterward, we integrated QTL meta-analysis results (Q < 0.05) with the Glioma International Case Control Study (GICC) GWAS meta-analysis (case:12,496, control:18,190), using a summary statistics-based mendelian randomization (SMR) method.

**Results:**

Between CMC and GTEx, we combined the QTL data of 354 unique individuals of European ancestry. SMR analyses revealed 15 eQTLs in 11 loci and 32 sQTLs in 9 loci relevant to glioma risk. Two loci only harbored sQTLs (1q44 and 16p13.3). In seven loci, both eQTL and sQTL coexisted (2q33.3, 7p11.2, 11q23.3 15q24.2, 16p12.1, 20q13.33, and 22q13.1), but the target genes were different for five of these seven loci. Three eQTL loci (9p21.3, 20q13.33, and 22q13.1) and 4 sQTL loci (11q23.3, 16p13.3, 16q12.1, and 20q13.33) harbored multiple target genes. Eight target genes of sQTLs (*C2orf80*, *SEC61G*, *TMEM25*, *PHLDB1*, *RP11-161M6.2*, *HEATR3*, *RTEL1-TNFRSF6B*, and *LIME1*) had multiple alternatively spliced transcripts.

**Conclusion:**

Our study revealed that the regulation of transcriptome by glioma risk alleles is complex, with the potential for eQTL and sQTL jointly affecting gliomagenesis in risk loci. QTLs of many loci involved multiple target genes, some of which were specific to alternative splicing. Therefore, quantitative trait loci that evaluate only total gene expression will miss many important target genes.

## Background

Gliomas are among one of the most devastating of rare cancers and are ranked first among all cancers in terms of average years of life lost (Rouse et al., 2016). The only environmental risk factor consistently identified is ionizing radiation (Ostrom et al., 2015). In the past decade, a number of genome-wide association studies (GWAS) and a meta-analysis of GWAS validated 25 risk alleles for glioma (Shete et al., 2009; Wrensch et al., 2009; Sanson et al., 2011; Stacey et al., 2011; Jenkins et al., 2012; Rajaraman et al., 2012; Enciso-Mora et al., 2013; Walsh et al., 2014; Kinnersley et al., 2015; Melin et al., 2017). The molecular mechanism of glioma risks conferred by most of these variants is unknown. A method to discover target genes of risk SNPs is molecular quantitative trait loci (molQTL) mapping, using molecular and single-nucleotide polymorphism (SNP) data from relevant cells and tissues for that trait. Although the ascertainment of relevant tissues for some traits had yielded surprising results, heritability enrichment analyses confirmed non-diseased brain tissues as the most relevant for diseases of the brain (Finucane et al., 2018; Hormozdiari et al., 2018).

Previous analyses integrating expression quantitative trait loci QTL (eQTL) data with glioma GWAS have provided limited insights to date. One study used glioma datasets from The Cancer Genome Atlas (TCGA) and lymphoblastoid cell line data from Genetic EUropean VAriation in DISease (GEUVADIS) (Lappalainen et al., 2013; Kinnersley et al., 2015); two others utilized brain tissue data of Genotype-Tissue Expression Project (GTEx), and one also used a blood eQTL dataset (Westra et al., 2013; Melin et al., 2017; Wu et al., 2019). Among the three studies, significant target genes were identified in three loci using GTEx brain tissues but none using glioma tissues from TCGA (Kinnersley et al., 2015; Melin et al., 2017). Two more loci harbored significant eQTL using genotyping and expression data from whole blood, but significance of the results was unclear because lymphocytes may not share heritability with central nervous system (CNS) cells. Therefore, there is a need of a larger QTL study or meta-analysis of QTLs based upon non-diseased brain tissues.

Moreover, the scope of eQTL analyses published to date was limited, because analyses often involved a limited number of correlated regulatory SNPs in each locus and *cis* gene. Recent functional assays suggested that few of the top GWAS risk alleles are themselves functional or causal, and many are in fact in linkage disequilibrium (LD) with one or several functional SNPs in a given locus (Biancolella et al., 2014; Fortini et al., 2014; Lawrenson et al., 2016; Buckley et al., 2019). Moreover, studies have found that target genes may not be the nearest gene to a risk SNP (Buxton et al., 2019; Farashi et al., 2019). Thus, there is a need for a more comprehensive QTL evaluation.

Approximately 48% of GWAS loci harbored eQTLs (Joehanes et al., 2017). For those without eQTLs, the effect of risk alleles may be mediated through alternative transcript splicing rather than total gene expression. In fact, RNA splicing is the most abundant within the brain (Yeo et al., 2004; Vuong et al., 2016). It plays an important role in normal function and development of the central nervous system (Vuong et al., 2016). Numerous studies have demonstrated that *de novo* germline mutations and germline variants contribute to the risk of neurological diseases by affecting alternative splicing (Xiong et al., 2015; Reble et al., 2018). Recently, two independent genome-wide splicing QTL (sQTL) mappings identified 8,966 and 9,028 sQTLs in the non-diseased human brain involving > 3,000 genes, respectively, supporting the idea that genetic variants commonly regulate gene transcription in the brain via the formation of alternatively spliced transcripts (Takata et al., 2017; Raj et al., 2018). Moreover, recent sQTL evaluations have contributed additional target genes of cancer risk variants (Gusev et al., 2019; Guo et al., 2020). Therefore, adding sQTL to eQTL analysis may lead to the discovery of alternative functional mechanisms not explained by eQTL study alone.

Here, we sought to evaluate sQTLs in validated glioma risk loci and to perform comprehensive eQTL and sQTL meta-analyses. To our knowledge, no prior study evaluated alternative spliced genes in glioma susceptibility. For this purpose, we used a validated, annotation-free quantification of the RNA splicing method to identify variable splicing events from short-read RNA-seq data. Moreover, to conduct both eQTL and sQTL meta-analyses, we pooled genotyping or whole-genome sequencing and RNA-seq data from the CommonMind Consortium (CMC), which is the largest resource of postmortem brain tissues in the United States (US), and GTEx (multiple brain tissues) for a combined sample size of 354 unique non-diseased individuals’ brain tissues (European ancestry) (Fromer et al., 2016; GTEx Consortium et al., 2017). In order to ensure that the same variants are likely responsible for the signals in both GWAS and QTLs, significant results from QTL meta-analyses were integrated with the Glioma International Case-Control Study (GICC) GWAS meta-analysis (Melin et al., 2017), using a summary data-based mendelian randomization method (SMR). Furthermore, we evaluated functional enrichment of significant SNPs identified through meta-analyses and SMR and further annotated SMR-associated SNPs with publicly available ChIP-seq and RNA-binding protein datasets.

## Materials and Methods

### Study Datasets

We used SNP genotyping and RNA-seq data from the CommonMind Consortium (CMC release 1.0) and Genotype-Tissue Expression Program (GTEx version 7.p2) for this meta-analysis (Fromer et al., 2016; GTEx Consortium et al., 2017). Data generated for CMC came from postmortem human brain specimens originating from the tissue collections at three brain banks: Mount Sinai NIH Brain Bank and Tissue Repository, The University of Pittsburgh Brain Tissue Donation Program, and the University of Pennsylvania Brain Bank of Psychiatric Illness and Alzheimer’s Disease Core Center (Fromer et al., 2016). We used only RNA-seq and genotyping data from 279 unique individuals (dorsal lateral prefrontal cortex), who did not have neuropsychiatric diseases or neurological insults immediately before death. Approval was obtained from the National Institute of Mental Health (NIMH) repository and genomic resources. The GTEx project was established to characterize human transcriptomes within and across individuals for a wide variety of primary tissues and cell types (GTEx Consortium et al., 2017). Since > 90% of glioma arise from the supratentorial compartment of the brain (Larjavaara et al., 2007), we chose the RNA-seq and SNP data of eight supratentorial non-diseased brain tissues (anterior cingulate cortex, caudate nucleus, cortex, frontal cortex, hippocampus, hypothalamus, nucleus accumbens, and putamen) from 190 unique individuals for our analyses. Approval was obtained from dbGAP.

### Genotyping and Whole-Genome Sequencing Data

CommonMind Consortium used the Infinium HumanOmniExpressExome v1.1 DNA Analysis Kit (Illumina, 958,178 SNPs) for genotyping. Following exclusion of those SNPs with genotyping call rate < 0.95, Hardy–Weinberg *P*-value < 5 × 10^–5^, and those without alternate alleles, we used Admixture v1.3.0 to ascertain ancestry and retained the samples of 216 unique individuals of European ancestry (Supplementary Table 1). We then performed imputation using the Sanger Imputation Server, with the UK10K and 1000 Genomes Phase 3 dataset as reference panels (1000 Genomes Project Consortium et al., 2015; UK10K Consortium et al., 2015). Those imputed SNPs with info score ≥ 0.5 were kept.

For the GTEx Consortium data (v7 release), we extracted SNPs from the genotype cell VCF file of whole-genome sequencing data. There were 10,526,813 SNPs at MAF ≥ 0.01. Quality control (QC) processes and admixture ascertainment were the same as those of the CMC datasets. Following QC and admixture analysis, we retained the genotyping data of 138 unique individuals of European ancestry for all subsequent QTL analyses (Supplementary Table 1).

To compile a complete list of candidate regulatory SNPs (or candidate functional SNPs) within the 25 glioma loci for eQTL and sQTL mapping, we empirically defined the SNP regions of interest as those localized ± 1.1 Mb from the top GWAS meta-analysis SNPs; candidate SNPs must have an *r*^2^ ≥ 0.2 with those top SNPs. The distance of 1.1 Mb was chosen because prior study had identified the 99th percentile of distance between SNP and target gene to be approximately 1.1 Mb (Thibodeau et al., 2015). Altogether, we extracted 4074 SNPs among the 25 loci. Moreover, we retrieved an additional 394 SNPs with *P*-values ≤ 1 × 10^–8^ in the fine mapping analysis of the GICC GWAS meta-analysis that have not yet been included among the 4074 SNPs (Melin et al., 2017). Some of these GWAS SNPs had *r*^2^ < 0.2 with the top GWAS SNPs. Therefore, the final number of candidate SNPs for both QTL mapping was a total of 4468 (Supplementary Figure 1).

### RNA-Seq Data

CommonMind Consortium (release 1) RNA-seq data (*N* = 216) were available as BAM files, which contained 100-bp paired-end reads and ≥50 million total reads per sample (Fromer et al., 2016). Sequencing was performed by using HiSeq2500 (Illumina). Sequencing libraries were prepared using rRNA depletion procedures. Downloaded BAM files for mapped and unmapped reads were merged by using SAMtools. Merged BAM files were converted into the FASTQ format using bam2fastq function within SAMtools.

We downloaded GTEx (v7) RNA-seq data from dbGAP as SRA files and converted them to FASTQ files using the SRA toolkit. Sequencing was performed using HiSeq 2500, which generated 76-bp paired-end reads and ≥50 million reads. We used a total of 670 FASTQ files from 138 unique individuals.

We processed FASTQ files from both CMC and GTEx using the same pipeline. We mapped reads from FASTQ files to hg19 using STAR v2.6 (two pass mode), with GENCODE V19 as the reference annotation (Dobin et al., 2013). FeatureCounts was used to generate the gene-level counts from the aligned reads (Liao et al., 2014). Quality control measures consisted of the criteria that all genes had >5 reads across all samples and <10 samples per gene had zero reads. Among the 25 loci (located ± 1.1 Mb from top GWAS SNPs), a total of 1559 met the criteria and were retained in both datasets for eQTL mapping. After quality control measures, we normalized and variance stabilized gene counts using DESeq2 (Love et al., 2014).

### Alternative RNA Splicing Quantification

To prepare for sQTL mapping, alternative transcripts were first evaluated using LeafCutter, which is an established method that used short-read RNA-seq data to detect intron excision events at base-pair precision by analyzing mapped split reads (Li et al., 2018). LeafCutter’s intron-centric view of splicing is based on the observation that mRNA splicing occurs predominantly through the step-wise removal of introns from nascent pre-mRNA. The advantage of this method is that it does not require read assembly or inference of isoforms supported by ambiguous reads, both of which are computationally and statistically difficult. The detailed method has been discussed elsewhere (Raj et al., 2018). Briefly, LeafCutter pools all mapped reads, finds overlapping introns demarcated by split reads, and then constructs a graph that connects all overlapping introns sharing a donor or acceptor splice site. The connected components of the graph form intron clusters, which represented alternative intron excision events. LeafCutter iteratively applies a filtering step to remove rarely used introns, which are defined on the basis of the proportion of reads supporting a given intron compared with other introns in the same cluster, and re-clusters leftover introns. Across the 25 validated loci, there were a total of 518 target genes with 6326 alternatively spliced intron clusters that formed the basis of sQTL mapping.

### eQTL Mapping

We performed eQTL analyses with covariates including age, sex, RIN scores, top three genotyping principal components, and 20 Probabilistic Estimation of Expression Residuals (PEER) factors, which were calculated from the normalized RNA expression matrices (Stegle et al., 2012). We used Matrix eQTL (v2.1.0) and multilevel linear regression with random intercept (lme4 package in R v3.5) for eQTL analysis of the CMC and GTEx datasets, respectively (Shabalin, 2012). Matrix eQTL does not accommodate multiple correlated brain tissue expression data from the same individuals; therefore, multilevel linear regression was used for the analysis of GTEx eQTL data. We adjusted for false discovery rate using the Benjamin–Hochberg method (FDR *Q* < 0.05).

### sQTL Mapping

We focused on the SNP–intron cluster that was within the ±100-kb window, as prior studies had reported that sQTL are mostly concentrated within this genomic distance (Takata et al., 2017; Raj et al., 2018). For sQTL identification, LeafCutter found alternatively excised intron clusters and quantified intron excision levels in all samples (Li et al., 2018). It then outputted intron excision proportions, which was the number of reads supporting a specific intron excision event to the total number of reads from that intron cluster. The ratios were then quantile normalized and used as input for Matrix eQTL and multilevel linear regression in sQTL analyses of CMC and GTEx, respectively. Covariates used were the same as eQTL mapping. To adjust for false discovery rates, the number of intron excision events in a cluster was first adjusted by Bonferroni correction, and subsequently FDR correction (FDR *Q* < 0.05) was applied for the number of sQTL per intron cluster. This 2-step multiple-comparison adjustment method was recommended for sQTL mapping with Leafcutter (Li et al., 2018).

### eQTL and sQTL Shared Between CMC and GTEx

To evaluate the sharing of eQTL and sQTLs between the two datasets, we used Storey’s QVALUE software implemented in R package (Storey and Tibshirani, 2003). The program takes a list of p-values and computes their estimated π_0_, which is the proportion of features that are truly null. Then, the quantity π_1_ = 1 - π_0_ estimates the proportion of true positives (TP). Sharing between two samples was reported as the proportion of TP estimated from the p-value distribution of independent QTLs discovered in the CMC dataset that is also present in the GTEx dataset.

### Meta-Analyses of eQTL and sQTL

For both eQTL and sQTL meta-analyses, we used the fixed-effect inverse variance weighted model to combine summary statistics (β and standard errors) of the CMC and GTEx. We used the *I*^2^ statistics to quantify the percentage of variation across studies that is due to heterogeneity rather than chance and is inherently not dependent upon the number of studies considered. We took the *I*^2^ value ≥ 75 to indicate significant heterogeneity. The mean overall *I*^2^ for all eQTL sand sQTLs were 0%, indicating overall low level of heterogeneity. We conducted all meta-analyses using METAFOR (v2.4) in R.

eQTL meta-analysis was considered significant at FDR *Q* < 0.05. For sQTL meta-analysis, the same two-level multiple-testing adjustment (described above) was applied.

### Integration of QTL Mapping and GWAS Data Using SMR Analyses

We integrated significant eQTL and sQTL meta-analysis results with the GICC GWAS meta-analysis (case:12,496, control:18,190) using summary-data-based mendelian randomization (SMR) analysis (Zhu et al., 2016; Melin et al., 2017). Approval for the GICC GWAS meta-analysis was obtained from the European Genome–phenome Archive (EGA). This SMR method assumed only one causal variant (affecting both probes and a trait) in any given locus; therefore, it tested the association between a trait and probes using the effect size of the top associated *cis*-QTLs from eQTL and sQTL mappings. A probe for eQTL was each significant target gene and for sQTL the individual intron cluster region. The input for the SMR analyses were significant QTL meta-analysis probes at FDR < 0.05 and GWAS SNPs with associated *P*-value < 5.0E-05. The 1000 Genome Project phase 3 data was used as the reference sample for LD estimation and allele frequency calculation (1000 Genomes Project Consortium et al., 2015). According to the SMR methodology, significantly colocalized QTLs must pass a pSMR threshold that is based upon Bonferroni correction of the total number of probes tested, which equated to the total number of target genes (eQTL) and total number of intron cluster regions (sQTL), as well as a pHEIDI > 0.05 without multiple-testing correction (Zhu et al., 2016). For eQTL, the pSMR threshold was 8.20E-04 for 61 probes, and for sQTL, the threshold was 4.81E-04 for 104 probes.

### Conditional Analyses

To find secondary QTL mapping signals, we performed conditional analyses using Genome-wide Complex Trait Analysis (GCTA) in the *cis*-QTL regions, condition on the top *cis*-eQTL or sQTL for significant probes found by SMR analyses (Yang et al., 2012). We used our QTL meta-analysis summary statistics data of GICC GWAS meta-analysis SNPs (*P* < 5.0E^–05^) for this purpose.

### Enrichment Analyses of eQTL and sQTL SNPs Within Epigenomic Marks and RNA Protein (RBP)-Binding Sites

We evaluated the enrichment within epigenomic marks and RBP-binding sites of our significant eQTL and sQTL meta-analysis SNPs and SMR-associated SNPs. We carried out the enrichment analyses using GREGOR (Genomic Regulatory Elements and GWAS Overlap Algorithm) v1.3.1 (Schmidt et al., 2015). For epigenomic marks, we retrieved the following publicly available ChIP-seq data: H3K4me1, H3Kme3, H3K27ac, H3K9me3, H3K27me3, H3K36me3, and DHS from Roadmap Epigenome Consortium and NCBI GEO. ChIP-seq datasets of the following cell types were considered: normal astrocytes, GBM stem cells, neural stem cells, H9 cells, H9-derived neuronal progenitor cells, and GBM cell lines (Supplementary Table 2). For RBP sites, we downloaded all sites from cross-linking immunoprecipitation (CLIP)-seq data of 171 human RBP collected within the CLIPdb database and 371 from MotifMapRNA (Yang et al., 2015; Liu et al., 2017). Files were converted to BED format and concatenated together as a single annotation file before analyses.

GREGOR evaluates the significance of the observed overlap by estimating the probability of the observed overlap of our input SNPs relative to expectation using a set of matched control variants. The control variants are random control SNPs selected across the genome that match the input SNPs based upon the number of variants in LD, minor allele frequency, and distance to nearest genes or intron clusters. The *P*-value calculated by GREGOR assumed a sum of binomial distributions to represent the number of index SNPs that overlap a dataset compared to the expectation observed in the matched control sets. In addition, we adjusted the *P*-value using FDR (significance is *Q* < 0.05) due to multiple testing.

### Functional Annotation and Visualization of SMR-Associated SNPs

We used SnpEff v4.3 to classify genomic positions of all SMR-associated SNPs (Cingolani et al., 2012). We further annotated these SNPs using the same set of publicly available ChIP-seq data as mentioned above for enrichment analyses. ChIP-seq data in BigWig files were aligned with LocusZoom plots in the UCSC genome browser to illustrate overlaps of SMR-associated SNPs within ChIP-seq peaks.

To evaluate the overlap of RNA-binding proteins (RBP) at genomic locations of SMR-associated SNPs for sQTL, we searched RBP-binding sites presented within CLIPdb and MotifMapRNA, as mentioned above (Yang et al., 2015; Liu et al., 2017). Furthermore, we used sQTLviztools (implemented in R) for visualization of sQTL (Tapial et al., 2017). Since LeafCutter does not provide a companion program for alternative transcript annotations, we overlapped the genomic coordinates of intron cluster regions with known alternatively spliced regions downloaded from VASTdb using the tool intersectBed within the BEDtools suite. Then, we identified known annotations using VAST tools (Tapial et al., 2017).

## Results

### eQTLs and sQTL Results of CMC and GTEx Datasets

To characterize the effect of glioma risk variants on gene regulatory processes in the brain, we performed a large-scale eQTL and sQTL scan in CMC and GTEx (Supplementary Figure 1). We tested a total of 4342 unique SNPs and 1559 target genes across 25 loci in eQTL, and 4342 unique SNPs, 518 spliced genes, and 6326 spliced intron cluster regions in sQTL analyses (Supplementary Figure 1). CMC and GTEx shared 1,859 significant eQTLs and 4,141 significant sQTLs (both at FDR *Q* < 0.05; Supplementary Tables 3, 4). We then estimated the degree of sharing of eQTLs and sQTLs between CMC and GTEx, using Storey’s π1 statistic (see section “Materials and Methods”). We found π1 = 0.74 for eQTLs and π1 = 0.84 for sQTLs, which suggested substantial sharing of eQTLs and sQTLs between these two independent datasets. The effect size was also highly correlated between the two datasets for shared eQTLs (Pearson *r* = 0.62, *P* = 3.24 E^–195^) and sQTLs (Pearson *r* = 0.91, *P* < 2.2 E^–16^). Taken together, these findings demonstrated that there was substantial concordance between significant sQTLs and eQTLs identified in CMC and GTEx.

### Meta-Analyses of eQTLs and sQTLs

We next performed a fixed-effect meta-analysis to maximize statistical power for eQTL/sQTL discovery. Our meta-analysis identified 5,943 significant eQTLs (FDR *Q* < 0.05) involving 66 target genes across 22 loci; there were 10,585 significant sQTLs (FDR *Q* < 0.05), involving 120 alternatively spliced intron cluster regions (i.e., alternatively spliced transcripts) within 28 target genes across 13 loci (Supplementary Tables 5, 6).

### Summary Data-Based Mendelian Randomization Analysis (SMR)

Following meta-analyses, we used SMR analysis which integrated summary-level data from GICC GWAS meta-analysis with significant eQTL and sQTL meta-analyses (FDR *Q* < 0.05; see section “Materials and Methods”). SMR revealed 15 eQTLs in 11 loci and 32 sQTLs in 9 loci that exceeded the predefined p-value threshold of the SMR test and passed the pHEIDI test (Tables 1, 2 and Supplementary Tables 7, 8). Therefore, the target genes and spliced genes in Tables 1, 2 are associated with glioma due to pleiotropy and are the most functionally relevant.

**TABLE 1:** Significant eQTLs using the SMR method: 15 SMR-associated eQTLs from 11 loci and their summary statistics.

Loci	GWAS SNP	SMR- associated SNP	A1/A2 (Co. Reg SNP)	A1 Freq (Co. Reg SNP)	*r*^2^ (Co. Reg SNP and GWAS SNP)	Target gene	β (SE) (GWAS)	*P*-value (GWAS)	β (SE) (eQTL)	*P*-value (eQTL)	*Q*-value (eQTL)
1p31.3	rs12752552	rs2780816	A/C	0.24	0.45	JAK1	–0.15 (0.03)	2.70E-08	–0.05 (0.01)	1.79E-07	4.34E-06
2q33.3	rs7572263	rs11883992	A/T	0.18	0.66	C2orf80	–0.19 (0.03)	5.73E-09	0.11 (0.02)	9.62E-07	6.89E-06
5p15.33	rs10069690	rs7712562	G/A	0.84	0.23	TERT	–0.40 (0.03)	2.09E-38	–0.15 (0.04)	1.68E-04	6.62E-03
7p11.2	rs723527 rs75061358	rs80013346	A/G	0.11	0.05 0.87	EGFR	0.45 (0.04)	6.48E-30	–0.26 (0.05)	2.55E-07	6.19E-05
9p21.3	rs634537	rs2106120	T/G	0.47	0.62	CDKN2B-AS1	–0.28 (0.02)	5.69E-36	0.11 (0.02)	2.23E-08	8.59E-07
		rs2518723	T/C	0.48	0.53	CDKN2B	0.24 (0.02)	7.02E-27	–0.12 (0.02)	2.36E-07	5.93E-06
10q24.33	rs11598018	rs10883948	T/G	0.54	1	RP11-541N10.3	–0.13 (0.02)	2.71E-08	0.09 (0.02)	3.57E-07	1.97E-06
11q23.3	rs12803321	rs573905	G/A	0.54	0.52	BCL9L	0.23 (0.02)	4.72E-21	–0.06 (0.02)	2.02E-04	1.52E-02
15q24.2	rs77633900	rs1875884	T/C	0.51	0.06	SCAPER	–0.13 (0.02)	2.63E-08	–0.06 (0.01)	8.91E-13	2.00E-10
16q12.1	rs10852606	rs8052492	G/A	0.29	0.99	HEATR3	–0.17 (0.02)	4.56E-12	–0.17 (0.01)	2.19E-34	3.89E-32
20q13.33	rs2297440	rs4809318	A/G	0.21	0.29	GMEB2	–0.25 (0.03)	1.56E-19	0.06 (0.01)	3.17E-05	1.06E-03
		rs6062497	T/C	0.71	0.49	ARFRP1	0.21 (0.02)	9.46E-18	0.05 (0.01)	4.21E-06	5.71E-04
		rs909334	A/C	0.21	0.62	STMN3	–0.28 (0.03)	1.77E-25	0.07 (0.02)	1.64E-06	4.35E-04
22q13.1	rs2235573	rs5756894	A/C	0.60	0.59	PICK1	–0.14 (0.02)	1.92E-09	–0.09 (0.01)	2.92E-09	5.36E-07
		rs6000991	C/T	0.61	0.57	SLC16A8	–0.13 (0.02)	1.05E-08	–0.15 (0.02)	1.76E-10	1.17E-08

**TABLE 2:** Significant sQTLs using the SMR method: 32 SMR-associated sQTLs from nine loci and their summary statistics.

Loci	GWAS SNP	SMR-associated SNP	A1/A2 (Co. Reg SNP)	A1 Freq (Co. Reg SNP)	*r*^2^ (Co. Reg SNP and GWAS SNP)	Target gene	Genomic location (hg19) of alternatively spliced intron cluster region from Leafcutter	β (SE) (GWAS)	*P*-value (GWAS)	β (SE) (sQTL)	*P*-value (sQTL)	*Q*-value (sQTL)
1q44	rs12076373	rs10927051	C/G	0.2	0.71	AKT3	clu_56564-243727150-243736228	–0.19 (0.03)	8.50E-10	0.3 (0.06)	1.26E-06	2.86E-04
2q33.3	rs7572263	rs7572263	G/A	0.24	1	C2orf80	clu_49792-209047771-209054677	–0.18 (0.03)	6.87E-10	–0.24 (0.04)	6.63E-10	1.62E-08
		rs7583625	G/A	0.25	0.96		clu_49792-209047771-209048618	–0.17 (0.03)	2.04E-09	–0.33 (0.03)	2.30E-22	7.05E-20
7p11.2	rs723527 rs75061358	rs2699247	A/G	0.36	0.03 0.07	SEC61G	clu_32096-54825287-54826851	0.14 (0.02)	6.62E-09	0.44 (0.06)	8.51E-13	1.70E-10
			A/G	0.36	0.03 0.07		clu_32096-54825287-54826855	0.14 (0.02)	6.62E-09	–0.39 (0.06)	7.23E-11	3.62E-09
11q23.3	rs12803321	rs11216924	G/C	0.14	0.22	TMEM25	clu_9404-118412790-118416834	–0.21 (0.04)	1.33E-08	0.33 (0.05)	8.51E-12	1.30E-09
		rs73023341	G/A	0.14	0.23		clu_9404-118405070-118419942	–0.21 (0.04)	1.35E-08	0.2 (0.04)	7.13E-08	1.17E-06
		rs61900957	T/C	0.09	0.16		clu_9432-118402586-118402865	–0.32 (0.04)	2.04E-14	–0.58 (0.1)	1.24E-09	2.84E-07
		rs11217021	C/T	0.25	0.43	DDX6	clu_9452-118627996-118630631	–0.21 (0.03)	1.25E-13	–0.17 (0.04)	6.12E-05	8.55E-03
		rs7125115	A/G	0.38	0.9	PHLDB1	clu_9440-118478414-118484531	–0.34 (0.03)	2.83E-23	–0.64 (0.05)	2.72E-35	1.06E-32
			A/G	0.38	0.9		clu_9440-118481241-118484009	–0.34 (0.03)	2.83E-23	0.43 (0.04)	4.63E-23	1.85E-21
			A/G	0.38	0.9		clu_9440-118481241-118484531	–0.34 (0.03)	2.83E-23	0.45 (0.05)	4.50E-22	1.30E-20
15q24.2	rs77633900	rs34213321	G/A	0.53	0.08	ETFA	clu_20348-76588078-76603691	–0.15 (0.02)	9.42E-10	0.36 (0.06)	1.36E-10	1.31E-09
16p13.3	rs3751667	rs34316274	G/A	0.23	0.75	LMF1	clu_16201-918097-918944	0.15 (0.03)	7.26E-08	–0.17 (0.04)	3.67E-06	1.31E-04
		rs34316274	G/A	0.23	0.75	RP11-161M6.2	clu_16093-1030705-1031145	0.15 (0.03)	7.26E-08	–0.3 (0.06)	4.21E-07	2.43E-06
		rs34316274	G/A	0.23	0.75		clu_16094-1026071-1026778	0.15 (0.03)	7.26E-08	0.31 (0.06)	1.87E-06	6.67E-06
		rs4984741	A/G	0.24	0.8		clu_16094-1026559-1026778	0.16 (0.03)	5.93E-09	0.24 (0.06)	1.26E-05	4.10E-05
16q12.1	rs10852606	rs12932038	C/T	0.71	1	HEATR3	clu_17583-50100180-50100278	0.17 (0.02)	5.47E-12	–0.34 (0.06)	8.69E-09	4.57E-06
		rs2058815	G/T	0.7	0.92		clu_17585-50109622-50109966	0.16 (0.02)	1.52E-10	–0.19 (0.04)	1.23E-06	1.64E-04
		rs2058815	G/T	0.7	0.92		clu_17585-50110000-50112652	0.16 (0.02)	1.52E-10	–0.17 (0.04)	1.96E-05	2.64E-04
		rs2287197	C/T	0.71	0.99		clu_17584-50102778-50103156	0.17 (0.02)	6.74E-12	–0.3 (0.04)	1.35E-15	5.16E-14
		rs2287197	C/T	0.71	0.99		clu_17584-50102778-50109482	0.17 (0.02)	6.74E-12	–0.33 (0.04)	1.09E-20	1.92E-18
		rs2287197	C/T	0.71	0.99		clu_17584-50103200-50104089	0.17 (0.02)	6.74E-12	–0.28 (0.04)	1.15E-13	2.23E-12
		rs8046344	G/C	0.71	1		clu_17584-50102778-50104055	0.17 (0.02)	1.42E-11	–0.21 (0.04)	1.17E-07	8.63E-07
20q13.33	rs2297440	rs1295810	A/G	0.2	0.78	RTEL1- TNFRSF6B	clu_30381-62321563-62321639	–0.35 (0.03)	4.86E-38	0.22 (0.04)	6.60E-10	3.32E-07
		rs2150910	C/T	0.91	0.25		clu_30376-62294908-62297357	0.37 (0.04)	1.33E-20	–0.35 (0.07)	1.18E-06	7.94E-04
		rs3208007	C/T	0.8	0.99		clu_30380-62320485-62320855	0.39 (0.03)	7.79E-46	0.66 (0.06)	7.90E-26	1.01E-24
		rs4809328	T/C	0.71	0.47		clu_30385-62325841-62326419	0.21 (0.02)	1.35E-17	0.21 (0.05)	4.88E-05	2.75E-02
		rs6062487	T/A	0.11	0.03	TNFRSF6B	clu_30388-62328544-62329633	0.35 (0.05)	6.97E-14	0.33 (0.08)	6.10E-05	1.96E-02
		rs6122154	T/C	0.19	0.75	LIME1	clu_30312-62367538-62368886	–0.34 (0.03)	2.62E-36	–0.23 (0.06)	1.41E-04	9.40E-04
			T/C	0.19	0.75		clu_30312-62368064-62368886	–0.34 (0.03)	2.62E-36	0.32 (0.07)	2.04E-06	1.52E-04
22q13.1	rs2235573	rs6000943	C/T	0.37	0.39	C22orf23	clu_28314-38341132-38343288	0.11 (0.02)	2.06E-06	0.24 (0.04)	1.10E-07	4.50E-05

We then performed conditional association analyses to evaluate the possibility of secondary QTL signals. Using Genome-wide Complex Trait Analysis (GCTA), condition on the top cis-eQTL or sQTL for significant probes identified through SMR analyses (those in Tables 1, 2), we did not find any secondary QTL signals.

Among loci with SMR-associated or colocalized eQTLs and sQTLs (Tables 1, 2), 1p31.3, 5p15.33, 9p21.3, and 10q24.33 only harbored eQTLs without sQTLs, whereas 1p44 and 16p13.3 had only sQTLs without eQTLs. In 7 loci (2q33.3, 7p11.2, 11q23.3, 15q24.2, 16p12.1, 20q13.33, and 22q13.1), eQTLs and sQTLs coexisted (Tables 1, 2), but the target genes were different for 5 of the 7 loci (7p11.2, 11q23.3, 15q24.2, 20q13.33, and 22q13.1). In the remaining two loci, 2q33.3 and 16q12.1, the target genes were the same.

Among the 11 loci with SMR-associated eQTLs, 9p21.3, 20q13.33, and 22q13.1 harbored multiple target genes (Table 1). The other 8 loci showed associations between single regulatory SNP and single target gene (Table 1). Similarly, among the nine loci with SMR-associated sQTLs, we found that four (1q44, 7p11.2, 15q24.2, and 22p13.1) harbored a single regulatory SNP associated with alternative splicing in a single gene (Table 2). In the remaining five loci (2q33.3, 16q12.1, 11q23.3, 16q13.3, and 20q13.33), there were evidence of multiple target genes with alternative splicing.

For sQTL target genes *SEC61G* (7p11.2), *PHLDB1* (11q23.3), and *LIME1* (20q13.33), a single regulatory SNP was associated with several intron cluster regions (or alternatively spliced transcripts) (Table 2), whereas multiple SNPs were associated with multiple alternatively spliced intron cluster regions for *C2orf80* (2q33.3), *RP11-161M6.2* (16p13.3), *HEATR3* (16q12.1), *TMEM25* (11q23.3), and *RTEL1-TNFRSF6B* (20q13.33) (Table 2).

### Enrichment Analyses of Meta-Analyses SNPs and SMR-Associated SNPs

We then evaluated whether significant QTL meta-analysis SNPs and SMR-associated SNPs were enriched with regulatory elements. Among meta-analysis SNPs, enrichment tests were significant (FDR *Q* values < 0.05) for DNase 1 hypersensitivity site (DHS), H3K36me3, H3K4me1, H3K4me3, H3K27ac, and H3K9me3 but not H3K27me3 for eQTL, whereas enrichment analyses were only positive for DHS and H3K36me3 for sQTL (Supplementary Table 9). In SMR-associated SNPs, only DHS enrichment was significant for eQTL and borderline significant for sQTL (Supplementary Table 9).

We also tested enrichment in RNA-binding protein (RBP) sites for SNPs associated with splicing QTLs. Similar to histone marks, meta-analysis SNPs were significantly enriched for RBP sites, but SMR-associated SNPs were not (Supplementary Table 9).

The lack of significance for histone marks and RBP site enrichments in SMR-associated SNPs may reflect low statistical power, because the total input SNPs were far fewer for those associated with SMR compared to the numbers in meta-analyses (Supplementary Table 9).

### Annotation of SMR-Associated SNPs

#### SMR-Associated SNPs in eQTL

**FIGURE 1:**
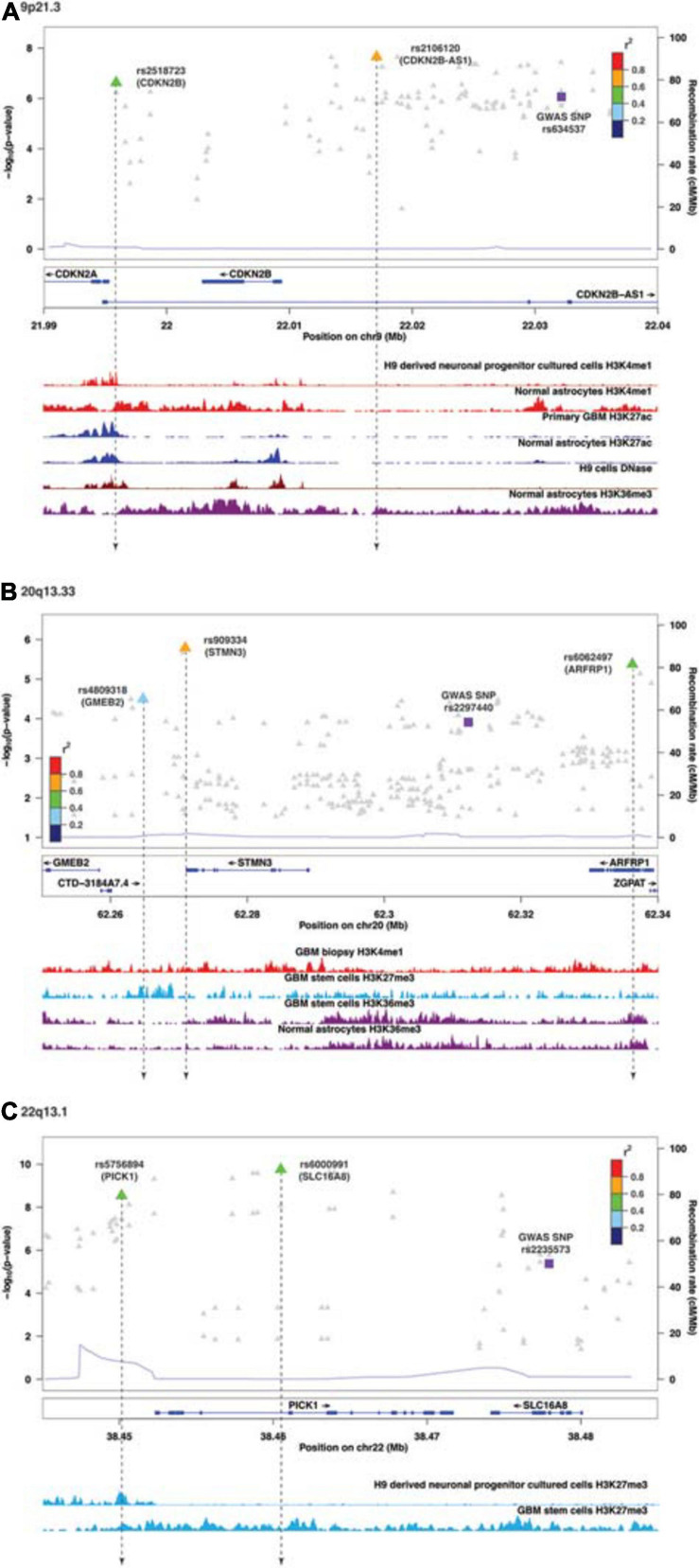
Visualization of SMR-associated eQTL SNPs and overlaps with ChIP-seq data. **(A)** 9p21.3, **(B)** 20q13.33, and **(C)** 22q13.1. The top panel shows the LocusZoom plot where the SNPs (triangles) are colored based on LD (*r*^2^) with the GWAS SNP (purple squares). The SNPs are labeled with the associated target genes in parentheses. The bottom panel shows the ChIP-seq peaks of epigenomic marks in various glioma or normal astrocytic cell lines; the individual ChIP-seq track was colored separately. The other significant SNPs (FDR *Q* < 0.05) that did not pass SMR tests are shown as gray dots in the background of the LocusZoom plot. SNPs that overlapped with ChIP-seq peaks are connected by a black dotted line.

Following enrichment analyses, we further annotated SMR-associated regulatory SNPs to four genomic locations: downstream (5 kb downstream of the most distal polyA addition site, *N* = 2), upstream (5 kb upstream of the most distal TSS, *N* = 4), intronic (*N* = 8), and intergenic (*N* = 1). Therefore, over half of eQTL SNPs were located within introns. Of the 11 SMR-associated eQTL loci, target genes within 9p21.3, 10q24.33, 20q13.33, and 22q13.1 were not the nearest genes to the colocalized SNPs. In 9p21.3, rs2518723 is located within an intron of *CDKN2B-AS1*, whereas the target gene is *CDKN2B* (Figure 1A); rs10883948 (10q24.33) is an intronic SNP within *STN-1*, but the target gene is the nearby lncRNA *RP11-541N10.3* (Supplementary Figure 2E). Likewise, rs6000991 (22q13.1) is located within an intron of *PICK1*, even though the target gene *SLC16A8* is located further telomeric (Figure 1C). In 20q13.33, eQTL SNP rs4809318 is located immediately telomeric to *CTD-3184A7.4*, but the target gene *GMEB2* is further centromeric from *CTD-3184A7.4* (Figure 1B). Therefore, similar to other reports from the literature, target genes of glioma risk variants may not be the closest one in genomic distance.

Functional annotations of SMR-associated SNPs using epigenomic marks showed that five eQTL SNPs resided within an enhancer (H3K4me1) and another five overlapped with a repressor (H3K27me3) (Table 3). Of the five that resided within an enhancer, four displayed a concomitant activation mark (H3K27ac) and two overlapped with open chromatins (DNase 1 hypersensitivity site) (Table 3). rs8052492 (16q12.1) was the only regulatory SNP located within an active promotor mark H3K4me3/H3K27ac and open chromatin. Other regulatory SNPs such as rs2106120 (9p21.3, *CDKN2B-AS1)* and rs6062497 (20q13.33, *ARFRP1*) overlapped with H3K36me3, whereas rs11883992 of 2q33.3 (*C2orf80*) overlapped with H3K9me3. The regulatory SNP rs10883948 of 10q24.33 is the only one that did not have any functional annotation using existing ChIP-seq datasets. Figures 1A–C illustrates the eQTL SNPs and their overlap with epigenomic marks for 9p21.3, 20q13.33, and 22q13.1. Supplementary Figures 2A–H showed the remaining eQTL loci.

**TABLE 3:** Functional annotations of significant SMR-associated eQTL SNPs.

Loci	SMR- associated SNP	Target gene	Genomic annotation (SnpEff)	Epigenomic annotation						
				
				H3K4me1	H3K4me3	H3K27ac	H3K9me3	H3K27me3	H3K36me3	DNase
1p31.3	rs2780816	JAK1	Intron	•		•				
2q33.3	rs11883992	C2orf80	Intron				•			
5p15.33	rs7712562	TERT	Upstream					•		
7p11.2	rs80013346	EGFR	Intergenic	•		•				
9p21.3	rs2106120	CDKN2B-AS1	Intron						•	
	rs2518723	CDKN2B	Intron	•		•				•
10q24.33	rs10883948	RP11-541N10.3	Intron							
11q23.3	rs573905	BCL9L	Intron					•		
15q24.2	rs1875884	SCAPER	Downstream	•		•				•
16q12.1	rs8052492	HEATR3	Upstream		•	•				•
20q13.33	rs4809318	GMEB2	Upstream					•		
	rs6062497	ARFRP1	Intron						•	
	rs909334	STMN3	Downstream	•						
22q13.1	rs5756894	PICK1	Upstream					•		
	rs6000991	SLC16A8	Intron					•		

#### SMR-Associated SNPs in sQTL

Likewise, a majority (17/23) of sQTL SNPs were also localized to introns, but unlike those of eQTLs, colocalized sQTL SNPs were also found within 3′UTR (2/23), 5′UTR (1/23), upstream (1/23), and exons (synonymous, 2/23). Moreover, we found that over half of them (12/23, 52.2%) overlapped with known RNA-binding proteins (Table 4).

**TABLE 4:** Functional annotations of significant SMR-associated sQTL SNPs.

Loci	SMR-associated SNP	Target gene	Genomic location (hg19) of alternatively spliced intron cluster region from Leafcutter	Alternative splicing event^$^	Genomic annotation (SnpEff)	RNA-binding proteins*	Epigenomic annotation						
	
							H3K4me1	H3K4me3	H3K27ac	H3K9me3	H3K27me3	H3K36me3	DNase
1q44	rs10927051	AKT3	clu_56564-243727150-243736228	ES	Intron	–				•			
2q33.3	rs7572263	C2orf80	clu_49792-209047771-209054677	ES	Intron	–	•		•				
	rs7583625		clu_49792-209047771-209048618		Intron	PTBP1, EIF4B	•		•				
7p11.2	rs2699247	SEC61G	clu_32096-54825287-54826851	Alt 5 SS	Intron	–	•		•				
			clu_32096-54825287-54826855	Alt 5 SS									
11q23.3	rs11216924	TMEM25	clu_9404-118412790-118416834	ES	Intron	RBM4B, HNRNPC						•	
	rs73023341		clu_9404-118405070-118419942	ES	Intron	–							
	rs61900957		clu_9432-118402586-118402865		Intron	RBM34						•	
	rs11217021	DDX6	clu_9452-118627996-118630631	ES	Intron	–						•	
	rs7125115	PHLDB1	clu_9440-118478414-118484531	ES	5’UTR	SRSF3, FUS		•	•				•
			clu_9440-118481241-118484009										
			clu_9440-118481241-118484531	ES									
15q24.2	rs34213321	ETFA	clu_20348-76588078-76603691	ES	Intron	–						•	
16p13.3	rs34316274	LMF1	clu_16201-918097-918944		Intron	A1CF, PUM1	•						
	rs34316274	RP11-161M6.2	clu_16093-1030705-1031145		Intron	A1CF, PUM1	•						
			clu_16094-1026071-1026778										
	rs4984741		clu_16094-1026559-1026778		Intron	ZNF368	•						
16q12.1	rs12932038	HEATR3	clu_17583-50100180-50100278		Intron	SNRPA, SRSF1							
	rs2058815		clu_17585-50109622-50109966		Upstream	–	•		•				
			clu_17585-50110000-50112652	Alt 5SS									
	rs2287197		clu_17584-50102778-50103156		Synonymous	–							
			clu_17584-50102778-50109482	ES									
			clu_17584-50103200-50104089										
	rs8046344		clu_17584-50102778-50104055	ES	Intron	–							
20q13.33	rs1295810	RTEL1- TNFRSF6B	clu_30381-62321563-62321639		3′UTR	SRSF4, B52	•		•				•
	rs2150910		clu_30376-62294908-62297357		3′UTR	CNOT4, ZNF638	•						•
	rs3208007		clu_30380-62320485-62320855		Synonymous	PPIG, DDX24							
	rs4809328		clu_30385-62325841-62326419	ES	Intron	-				•			
	rs6062487	TNFRSF6B	clu_30388-62328544-62329633		Intron	EFTUD2, RBM22						•	
	rs6122154	LIME1	clu_30312-62367538-62368886		Intron	SAMD4A, ACO1, EIF4B					•		
			clu_30312-62368064-62368886										
22q13.1	rs6000943	C22orf23	clu_28314-38341132-38343288		Intron	-						•	

*RNA-binding proteins: PTBP1, polypyrimidine tract-binding protein 1; EIF4B, eukaryotic translation initiation factor 4b; RBM4B, RNA-binding motif protein 4B; HNRNPC, heterogeneous nuclear ribonucleoproteins C1/C2; RBM34, RNA-binding protein 34; SRSF3, splicing factor arginine/serine-rich 3; FUS, FUS RNA-binding protein; A1CF, APOBEC1 complementation factor; PUM1, Pumilio RNA-binding family member 1; SNRPA, U1 small nuclear ribonucleoprotein A; SRSF1, serine/arginine-rich splicing factor 1; SRSF4, serine/arginine-rich splicing factor 4; B52, serine–arginine protein 55; CNOT4, CCR4-NOT transcription complex subunit 4; ZNF638, zinc finger protein 638; PPIG, peptidylprolyl isomerase G; DDX24, dead-box helicase 24; EFTUD2, elongation factor tu GTP-binding domain-containing 2; RBM22, RNA binding motif protein 22; SAMD4A, sterile alpha motif domain containing 4A; ACO1, aconitase 1. ^$^IR, intron retention; ES, exon skipping; Alt 5 SS, Alt. 5′ splice site.

**FIGURE 2:**
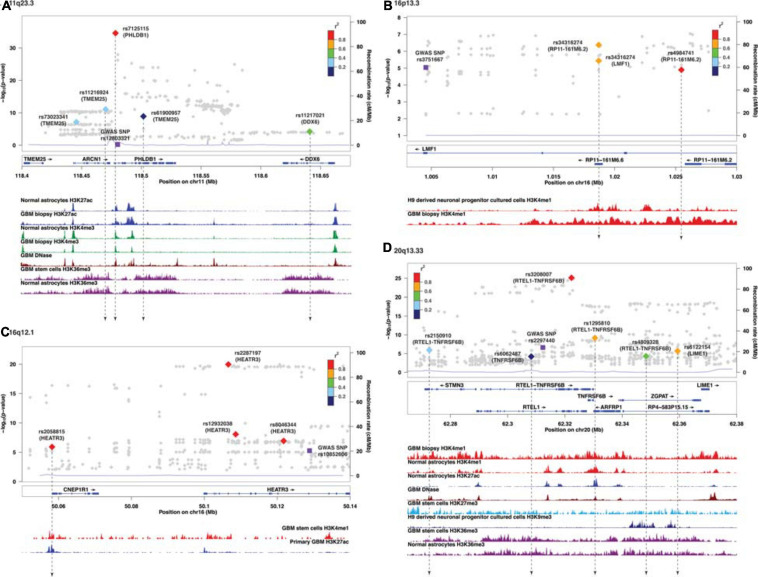
Visualization of SMR-associated sQTL SNPs and overlaps with ChIP-seq data. **(A)** 11q23.3, **(B)** 16p13.3, **(C)** 16q12.1, and **(D)** 20q13.33. The top panel shows the LocusZoom plot where the SNPs (triangles) are colored based on LD (*r*^2^) with the GWAS SNP (purple squares). The SNPs are labeled with the associated target genes in parenthesis. The bottom panel shows the ChIP-seq peaks of epigenomic marks in various glioma or normal astrocytic cell lines; the individual ChIP-seq track was colored separately. The other significant SNPs (FDR *Q* < 0.05) that did not pass SMR tests are shown as gray dots in the background of the LocusZoom plot. SNPs that overlapped with ChIP-seq peaks are connected by a black dotted line.

Similar to target genes of eQTLs, the target spliced genes were not always the closest in physical distance to the candidate functional SNPs, even though alternative splicing mediated by risk alleles is usually within 100 kb of the candidate functional SNPs (Takata et al., 2017; Raj et al., 2018). In six of nine sQTL loci, namely, 7p11.2, 11q23.3, 16p13.3, 16q12.1, 20q13.33, and 22q13.1, the target genes were not the nearest genes to the SMR-associated SNPs (Figures 2A–D and Supplementary Figures 3C,E). In 20q13.33, four SNPs regulated the alternative splicing of *RTEL-TNFSF6B*, but only one (rs3208007, exon-synonymous) was located within the target gene (Table 4). rs1295810 was situated within the 3′UTR of *ARFRP1* and rs4809328 within an intron of *ZGPAT*, which were telomeric to *RTEL1-TNFRSF6B*. The 4th SNP rs2150910 was within the 3′UTR of *STMN3*, which was centromeric to *RTEL1-TNFRSF6B* (Figure 2D). In *LIME1*, another target gene that was alternatively spliced in 20q13,33, the associated SNP rs6122154 resided within an intron of *ZGPAT*, which was centromeric to *LIME1* (Figure 2D).

In 11q23.3, rs11216924, rs73023341, and rs61900957 were the three SNPs associated with alternative splicing of *TMEM25* (Table 2). However, rs11216924 and rs73023341 were both intronic SNPs within *ARCN1*, whereas rs61900957 was an intronic SNP within *PHLDB1* (Figure 2A and Table 4). Both *ARCN1* and *PHLDB1* are genes telomeric to the target gene *TMEM25*. In 16p13.3, rs34316274 was localized within an intron of *LMF1* but mediated alternative splicing of the nearby lncRNA *RP11-161M6.2* as well as *LMF1* itself (Figure 2B and Table 4). Similarly, within 16q12.1, the closest gene to rs2058815 was *CNEP1R1*; nevertheless, it affected alternative splicing of *HEATR3*, which was further telomeric to *CNEP1R1* (Figure 2C and Table 4). In 22q13.1, rs6000943 is an intronic SNP within *MICALL1*; however, its effect was on the splicing of *C22orf23*, which was the target gene telomeric to it (Supplementary Figure 3E and Table 4). Lastly, rs2699247 in 7p11.2 affected the alternative transcription of *SEC61G* despite its location within an intron of the lncRNA *RP11-745C15.2* (Supplementary Figure 3C and Table 4).

Similar to SMR-associated eQTL SNPs, sQTL SNPs were commonly enriched with H3K4me1 (34.8%, 8/23), and five of the eight also overlapped with H3K27ac (Table 4). The second most common epigenomic mark for sQTL SNPs was H3K36me3 (26.1%, 6/23). Only rs7125115 (target gene *PHLDB1*) localized to both H3K4me3 and H3K27ac. Three sQTL SNPs did not show any histone mark occupancy: rs73023341 (*TMEM25*), rs2287197 (*HEATR3*), and rs3208007 (*RTEL1-TNFRSF6B*) (Table 4).

**FIGURE 3:**
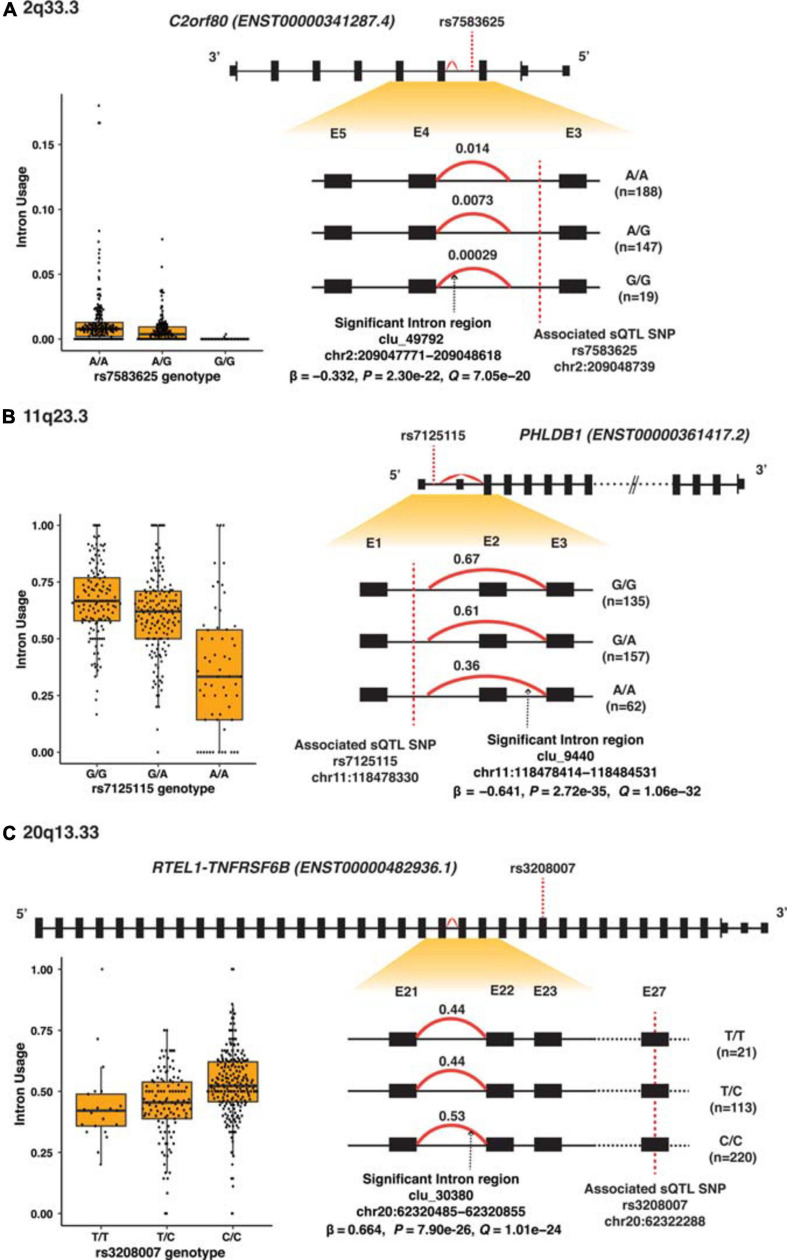
Splicing analyses of the three most significant sQTL. **(A)** 2q33.3 (*C2orf80*), **(B)** 11q23.3 (*PHLDB1*), and **(C)** 20q13.33 (*RTEL1-TNFRSF6B*). Top panel shows the high-level view of the gene: the black boxes represent exons; the smaller black boxes represent 5′ and 3′ UTRs and the connecting black lines represent introns. The gene structure is based on the primary transcript for each gene, and the size of the exons and introns is not according to the actual genomic region scale. The lower right panel shows the zoom-in view of the region of interest containing the alternatively spliced intron regions (red curve) and intron usage [percentage spliced in (PSI)] associated with each genotype; exons are labeled numerically and sequentially from 5′ to 3′. The lower left are box plots which showed PSI values against each of the sQTL SNP genotype.

Among the 32 SMR-associated sQTLs, 14 had known alternative splicing annotations (43.8%), and 11 of these 14 (78.6%) were exon skipping. The other three had alternative 5′ splicing (target genes *SEC61G* and *HEATR3*). Figure 3A–C illustrates differential splicing analyses by genotypes of the three highly significant sQTL: rs7583625 (*C2orf80*), rs7125115 (*PHLDB1* intron cluster 9440), and rs3208007 (*RTEL1-TNFRSF6B* intron cluster 30380). In each sQTL illustrated in Figure 3, the minor alleles were associated with decreased intron usage (PSI values). The differential splicing analyses of the rest of sQTL SNPs were illustrated in Supplementary Figures 4A–Z.

## Discussion

The finding of this study is the first to show that glioma GWAS risk alleles may mediate their effect through alternatively spliced transcripts. Two loci, 1p44 and 16q13.3, only harbored sQTLs, and an additional seven loci had coexisting eQTL and sQTLs. Furthermore, there were more SMR-associated sQTLs than eQTLs (32 sQTLs versus 15 eQTLs), which suggested that alternative splicing may be an important molecular mechanism in gliomagenesis mediated by GWAS risk alleles. Target genes were different between eQTL and sQTL for many of the loci with both types of QTL, which further illustrated the complexity of functional regulation of these risk loci (Gusev et al., 2019).

The most common type of alternative RNA splicing in the brain is skipped exon (Vuong et al., 2016; Reble et al., 2018), and the brain ranked the highest among 16 human tissues in terms of the proportion of genes with skipped exons (Yeo et al., 2004). This study found that 11 of the 14 known annotated sQTL involved skipped exons, which is concordant with what is known about the alternative splicing mechanism of the brain to date. Our finding also suggested that many of the sQTL SNPs associated with spliced RNA were within binding sites for RNA-binding proteins (RBPs), thus raising the possibility that these variants may alter the ability of RBPs to bind and interact with pre-mRNA and other RBPs within a spliceosome (Fu and Ares, 2014). Moreover, glioma risk variants may affect splicing through the process of trimethylation of H3K36 (Leung et al., 2019), and H3K36me3 is the second most common epigenomic mark overlapping with sQTL but not eQTL SNPs in this study. Methylation of H3K36 in the gene body (introns) has recently been revealed to be an important facilitator of spliceosome assembly, through its ability to recruit various adaptor proteins to support RNA splicing (Teissandier and Bourc’his, 2017; Leung et al., 2019). Furthermore, H3K36me3 has been associated with exon skipping (Shindo et al., 2013). Thus, glioma risk alleles may promote exon skipping through interference of the H3K36 trimethylation process and spliceosome assembly (Monteuuis et al., 2019).

There was no overlap between SMR-associated SNPs of eQTLs and sQTLs; moreover, with the exception of 2p33.3 (*C2orf80*) and 16q12.1 (*HEATR3*), the target genes were otherwise different for the remaining five loci with coexisting eQTLs and sQTLs. This suggests that sQTLs may act independent of eQTLs in mediating gliomagenesis. If quantitative trait loci mapping did not include splicing evaluation, a total of 12 of 26 (46.2%) target genes would have been missed, including two loci (1q44 and 16p13.3) which exclusively harbored sQTLs and no eQTLs.

Of all the target genes involved in sQTLs, none had previously known molecular mechanism mediated by alternative RNA splicing in glioma, although a number of the genes had been shown to be involved in glioma pathogenesis, progression, or prognosis. For example, *AKT3* (1q44) promotes glioma progression and represents a key resistance factor (Turner et al., 2015). *SEC61G* is a proto-oncogene required for glioblastoma cell survival (Lu et al., 2009). *DDX6* is involved in radio- and chemoresistance in glioblastoma (Cho et al., 2016), and *TNFRSF6B* suppresses CD95 ligand-induced apoptosis and chemotaxis in malignant glioma (Roth et al., 2001). The functional roles of the rest of spliced target genes have yet to be discovered. Of interest, *RTEL1* and the read-through transcript *RTEL1-TNFRSF6B* had been postulated to be target genes in 20q13.33 due to its role in telomere maintenance and also the fact that the top glioma GWAS risk allele resides within an intron of *RTEL1* (rs2297440) (Melin et al., 2017). However, no prior evaluation provided evidence that it is a significant eQTL target gene, and its biological role in the promotion and progression of glioblastoma has yet to be elucidated. This study found that *RTEL1-TNFRSF6B* is an sQTL but not an eQTL target gene, and three of the four SMR-associated sQTL SNPs were mapped outside of *RTEL1-TNFRSF6B*. Similarly, *PHLDB1* has long been speculated as the target gene in 11q23.3 due to the location of the top GWAS SNP which is within an intron of the gene (rs12803321) and its role in modulating AKT phosphorylation (Zhou et al., 2010), but this study found alternative splicing of the *PHLDB1* transcript to be the mechanism mediated by germline SNPs.

Among SMR-associated eQTL loci, 7p11.2, 11q23.3, 20q13.33, and 22q13.1 have coexisting sQTLs, but their target genes were different, suggesting that the regulation on gliomagenesis by these loci is more complex than previously realized. Among eQTL target genes, *BCL9L* (11q23.3), *SCAPER* (15q24.2), *RP11-541N10.3* (10q24.33) *CDKN2B-AS1* (9p21.3), and *C2orf80* (2q33.3) were newly discovered, replicated, and integrated with GWAS findings, and they have not been previously reported in eQTL analyses using non-diseased brain tissues. In 11q23.3, which is a locus associated with *IDH1*-mutated glioma, the hypothesized target gene had been *PHLDB1*. However, our finding showed that the gene is *BCL9L*, which is a transcription regulator associated with WNT signaling in glioma (Lee et al., 2016; Gay et al., 2019). In 15q24.2, the target gene *SCAPER* transcribes into a cyclin A-interacting protein which regulates cell cycle progression, but its role in gliomagenesis had not been previously evaluated (Tsang et al., 2007). *RP11-541N10.3* in 10q24.33 is a long non-coding RNA (lncRNA) with unknown function in glioma development. Within 2q33.3, which is also a locus associated with *IDH1*-mutated glioma (Kinnersley et al., 2018), the target gene *C2orf80* is 50 kb telomeric to *IDH1*, but whether or how *C2orf80* interacts with *IDH1* remains to be elucidated. In 9p21.3, a recent transcriptome-wide association study (TWAS) in glioma only found *CDKN2B* but not its anti-sense transcript as candidate causal genes (Atkins et al., 2019). To our knowledge, this study is the first to report *CDKN2B-AS1* (*ANRIL*), as well as *CDKN2B* as significant potential target genes in 9p21.3.

For the remaining eQTL target genes, this meta-analysis further validated the finding of two eQTL analyses (published as part of two previous GWAS studies) and one TWAS in glioma (Melin et al., 2017; Kinnersley et al., 2018; Atkins et al., 2019). They included *TERT* (5p15.33), *JAK1* (1p31.3), *EGFR* (7p11.2), *HEATR3* (16q12.1), *GMEB2, ARFRP1*, and *STMN3* (20q13.33), and *PICK1* and *SLC16A8* (22q13.1).

Common to other QTL mapping studies, a limitation of this study is the context of gene expression, which is limited to that of adult normal brain. If the manifestation of sQTL or eQTL is dynamic and only occurred during a certain developmental stage or early in gliomagenesis, this study is not set up to discover them. However, the advantage of using adult normal brain tissues is the ability to procure them through autopsy, and the relative confidence of isolating the influence of SNPs on gene expressions without the confounding effects of somatic alterations. We also acknowledged that bulk RNA-seq data consisted of a mixture of neurons and glial cells, but the single-cell eQTL dataset is uncommon and has less discovery power (6.9-fold differences) than similar-sized bulk RNA-seq QTL datasets (van der Wijst et al., 2018). Therefore, we aimed to leverage QTL meta-analyses using bulk RNA-seq for maximum power. Last, a limitation of using cell lines in ChIP-seq analysis is that it requires a large number of cells (>10^5^ cells), and the analysis focuses on average peak calling without accounting for heterogeneity between cells. However, the single-cell ChIP-seq dataset is rarely available, and the abundance of bulk ChIP-seq data from related cell types may allow for comparisons of functional elements.

In summary, this study identified alternative RNA splicing as a potential mechanism that may provide additional explanations for the functional basis of nine glioma risk loci. This study also showed that functional variants may influence total transcript abundance as well as spliced isoforms, and the target genes for eQTL and sQTL may differ in loci with both types of QTL. Finally, this meta-analysis identified comprehensively target genes that may serve as a reference for future functional assays.

## The Glioma International Case Control Study

Elizabeth B. Claus (School of Public Health, Yale University, New Haven, CT 06510, United States and Department of Neurosurgery, Brigham and Women’s Hospital, Boston, MA 02115, United States); Dora Il’yasova (Department of Epidemiology and Biostatistics, School of Public Health, Georgia State University, Atlanta, GA 30303, United States; Duke Cancer Institute, Duke University Medical Center, Durham, NC 27710, United States and Cancer Control and Prevention Program, Department of Community and Family Medicine, Duke University Medical Center, Durham, NC 27710, United States); Joellen Schildkraut (Department of Public Health Sciences, School of Medicine, University of Virginia, Charlottesville, VA 22903, United States); Jill S. Barnholtz-Sloan (Department of Population and Quantitative Health Sciences and the Cleveland Center for Health Outcomes Research, Case Western Reserve University School of Medicine, Cleveland, OH 44106, United States); Sara H. Olson (Department of Epidemiology and Biostatistics, Memorial Sloan Kettering Cancer Center, New York, NY 10017, United States); Jonine L. Bernstein (Department of Epidemiology and Biostatistics, Memorial Sloan Kettering Cancer Center, New York, NY 10017, United States); Christoffer Johansen (Danish Cancer Society Research Center, Survivorship, Danish Cancer Society, Copenhagen 2100, Denmark; ^15^Oncology Clinic, Finsen Centre, Rigshospitalet, University of Copenhagen, Copenhagen 2100, Denmark); Robert B. Jenkins (Department of Laboratory Medicine and Pathology, Mayo Clinic Comprehensive Cancer Center, Mayo Clinic, Rochester, MN 55905, United States); Beatrice S. Melin (Department of Radiation Sciences, Umeå University, Umeå 901 87, Sweden); Margaret R. Wrensch (Department of Neurological Surgery, School of Medicine, University of California, San Francisco, CA 94143, United States); Richard S. Houlston (Division of Molecular Pathology, The Institute of Cancer Research, London SW7 3RP, United Kingdom); Melissa L. Bondy (Department of Epidemiology and Population Health, Stanford Cancer Institute, Stanford University, Stanford, CA 94305, United States).

## Data Availability Statement

Publicly available datasets were analyzed in this study. This data can be found here: GICC GWAS summary statistics: EGA: https://www.ebi.ac.uk/ega/, accession number EGAS00001003372; GTEx: https://dbgap.ncbi.nlm.nih.gov/dbGaP, accession number: phs001319 v7; CMC: https://nimhgenetics.org/resources/commonmind# version 1.

## Author Contributions

RL designed the study. GICC Study provided the GWAS data. RL, DN, and CP analyzed the data and interpreted the results. RL, DN, and CP drafted the manuscript. All the authors reviewed and approved the manuscript.

## Conflict of Interest

The authors declare that the research was conducted in the absence of any commercial or financial relationships that could be construed as a potential conflict of interest.

## Supplementary Material

Supplementary Figure 1 Overall study schema. Workflow showing the analyses and results of individual datasets, meta-analysis and colocalization.

Supplementary Figure 2 Mapping of SMR associated eQTL SNPs, target genes and functional elements in loci with single target gene and single regulatory SNP: **(A)** 1p31.3, **(B)** 2q33.3, **(C)** Sp15.33, **(D)** 7p11.2, **(E)** 10q24.33, **(F)** 11q23.3, **(G)** 15q24.2, and **(H)** 16q12.1.

Supplementary Figure 3 Mapping of SMR associated sQTL SNPs, target genes and functional elements in the five remaining loci: **(A)** 1q44, **(B)** 2q33.3, **(C)** 7p11.2, **(D)** 15q24.2, and **(E)** 22q13.1.

Supplementary Figure 4 Alternative splicing analyses for the remaining 18 sQTLs (S4A-S4R). For the regulatory SNPs which reside in the neighboring target genes; the neighboring genes are shown in blue.
