# Supplementary material for: Meta-Analyses of Splicing and Expression Quantitative Trait Loci Identified Susceptibility Genes of Glioma
Source: Front Genet. 2021 Apr 15;12:609657. doi: 10.3389/fgene.2021.609657 (PMC8081720; doi:10.3389/fgene.2021.609657)

## Supplementary Figure 2A. 1p31.3

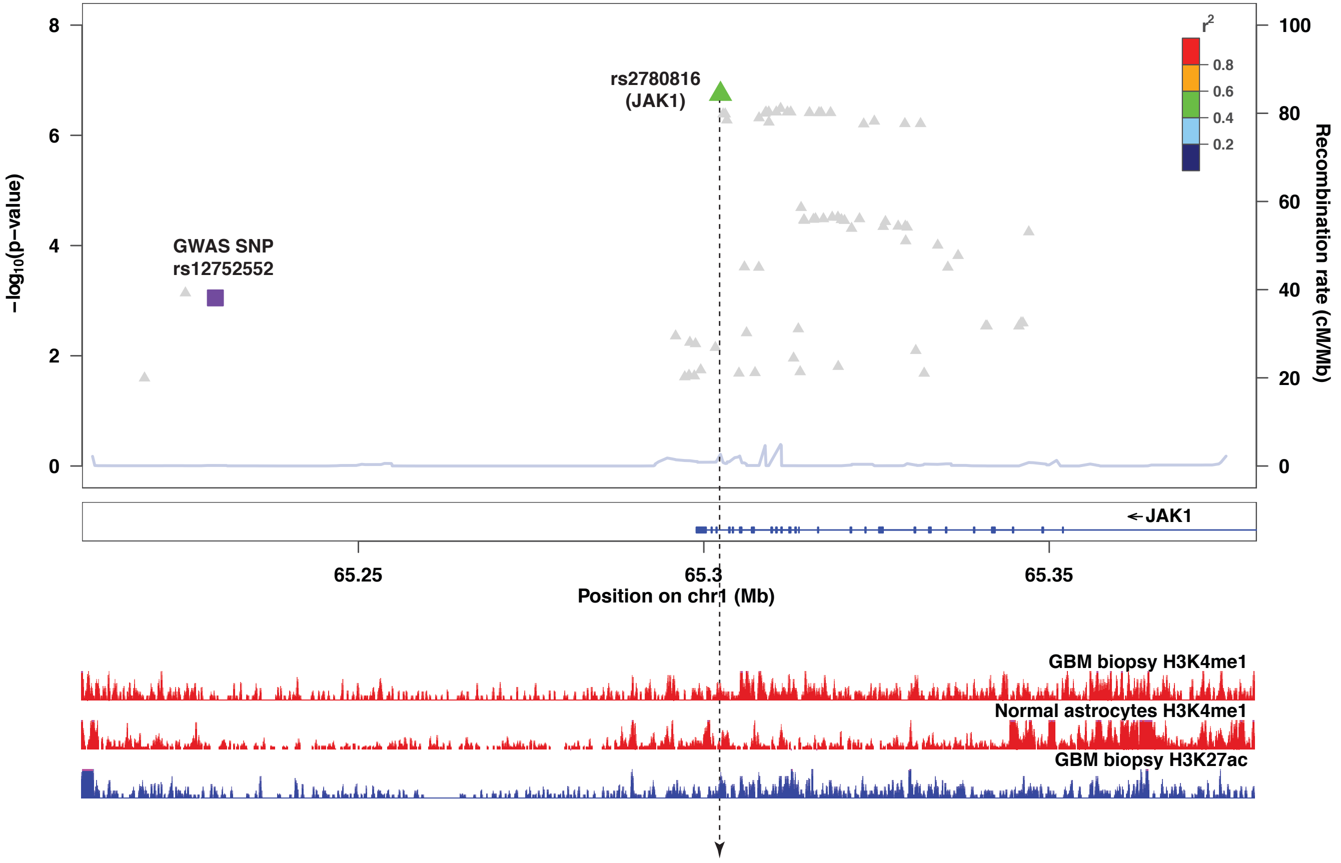

Supplementary Figure 2B. 2q33.3

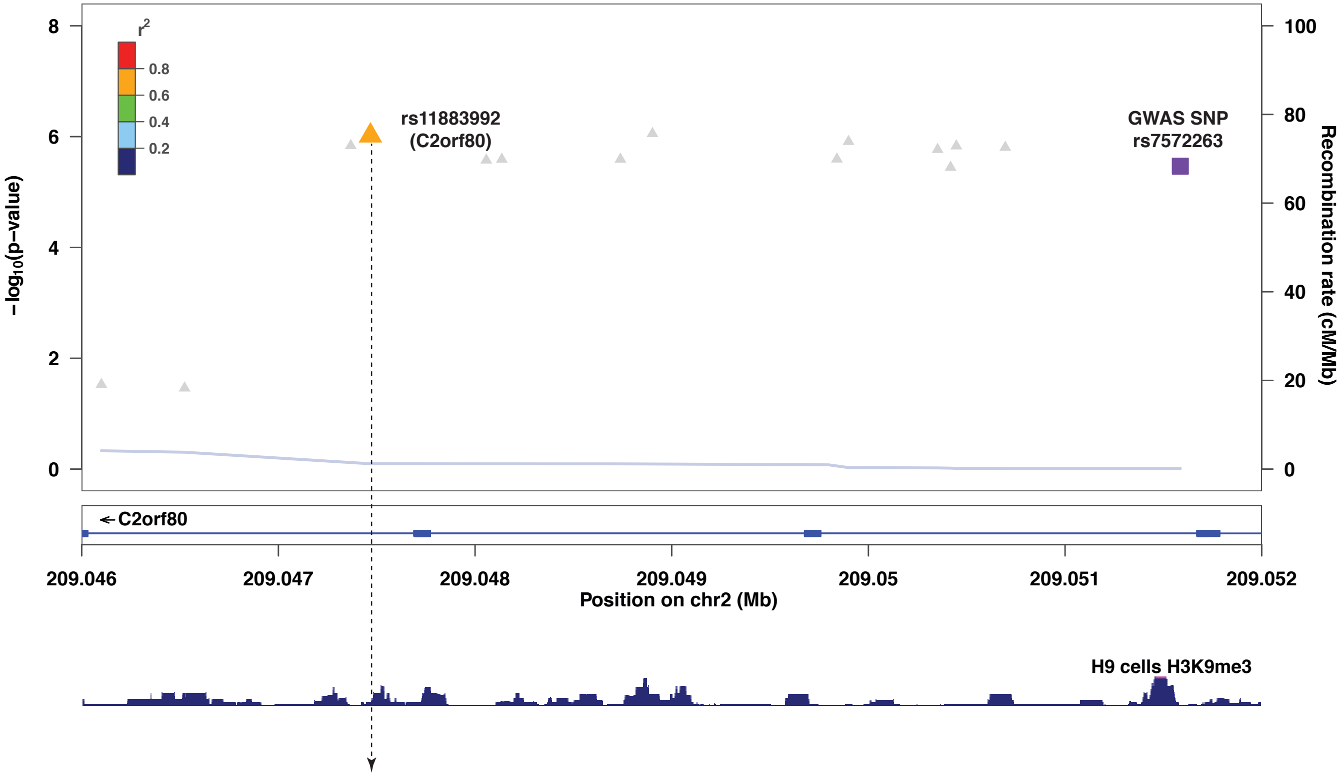

Supplementary Figure 2C. 5p15.33

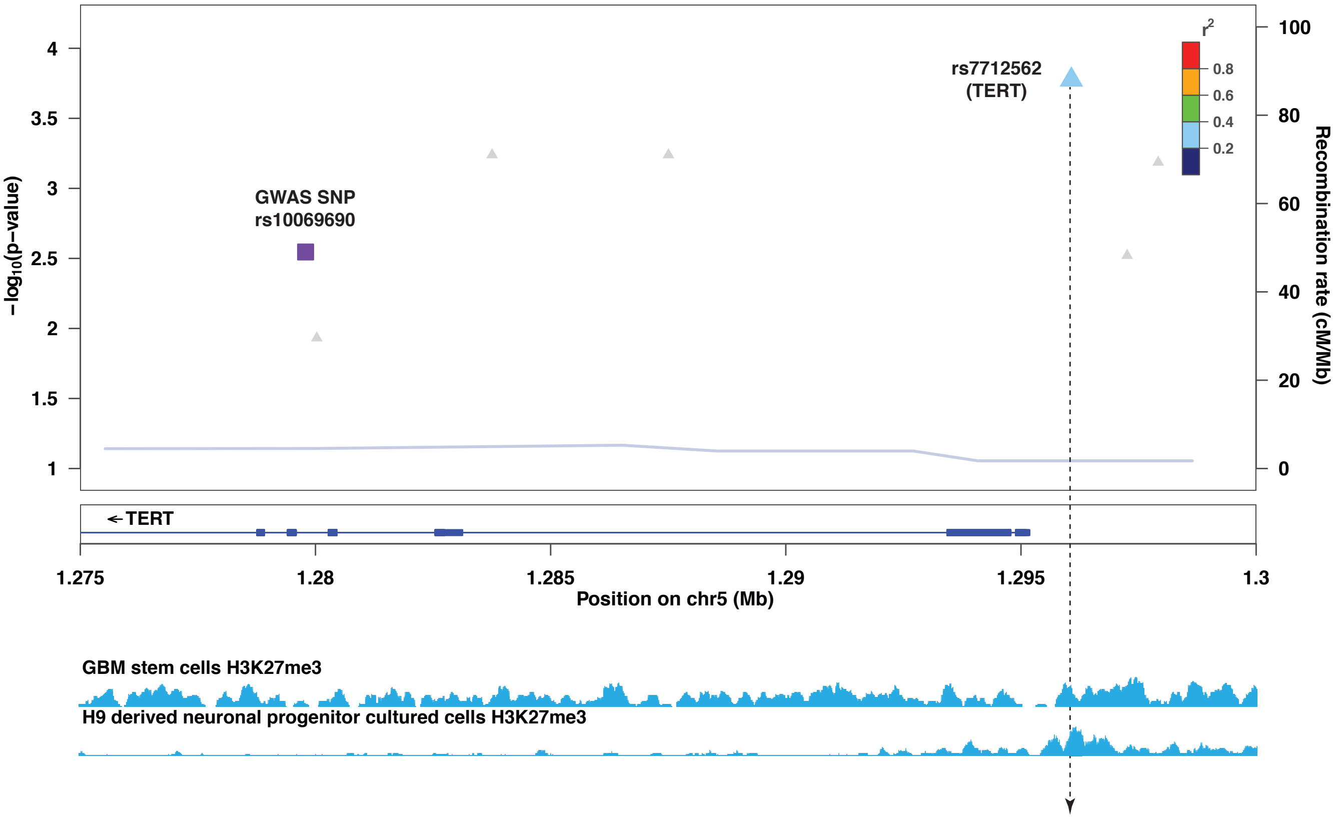

## Supplementary Figure 2D. 7p11.2

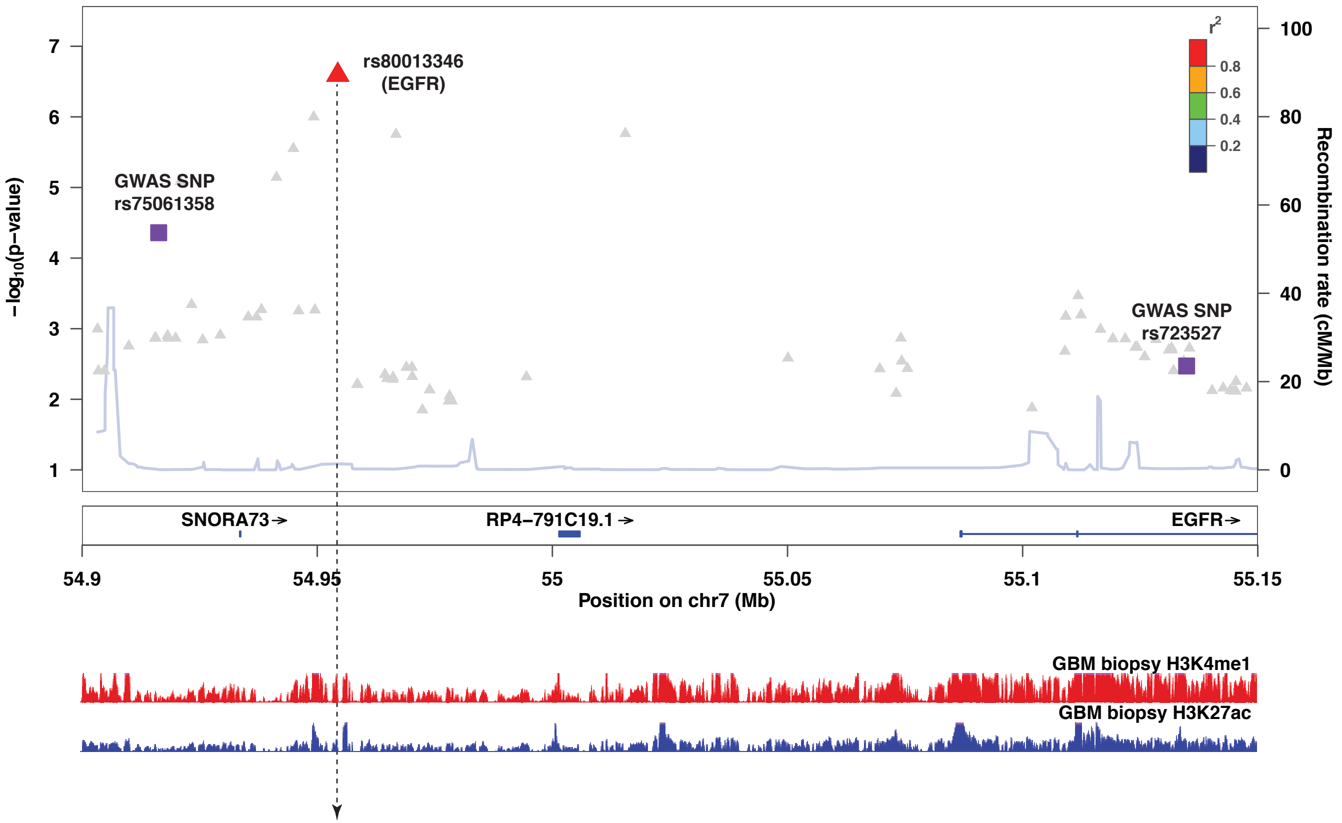

Supplementary Figure 2E. 10q24.33

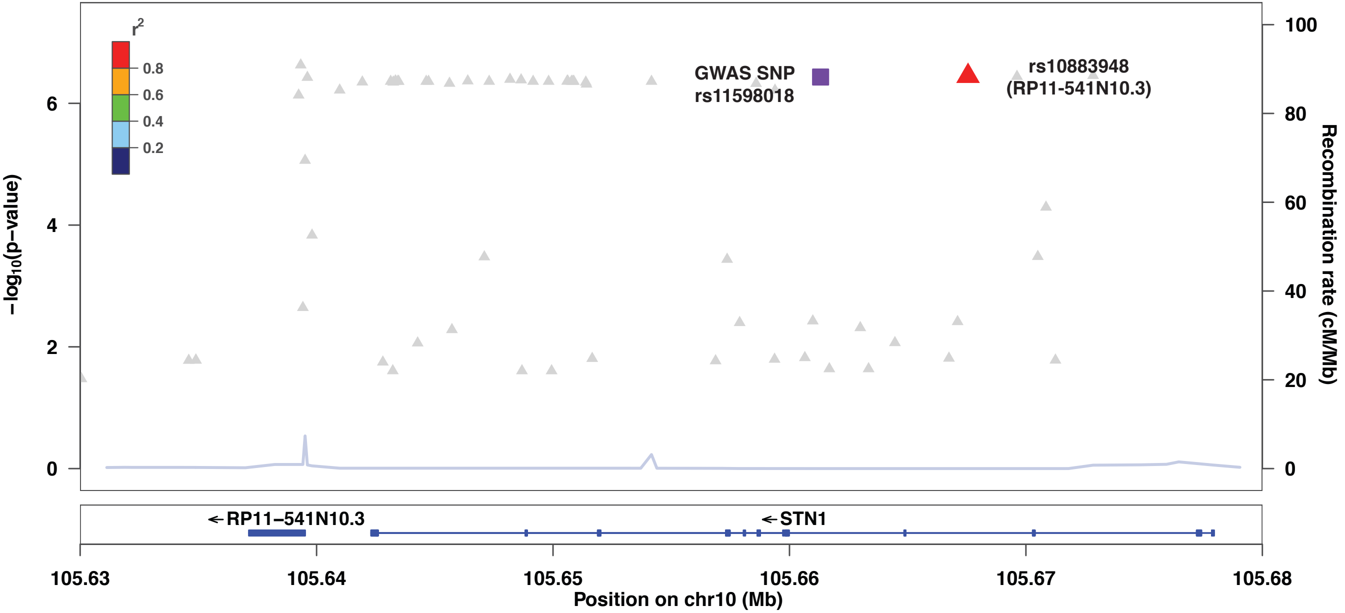

## Supplementary Figure 2F. 11q23.3

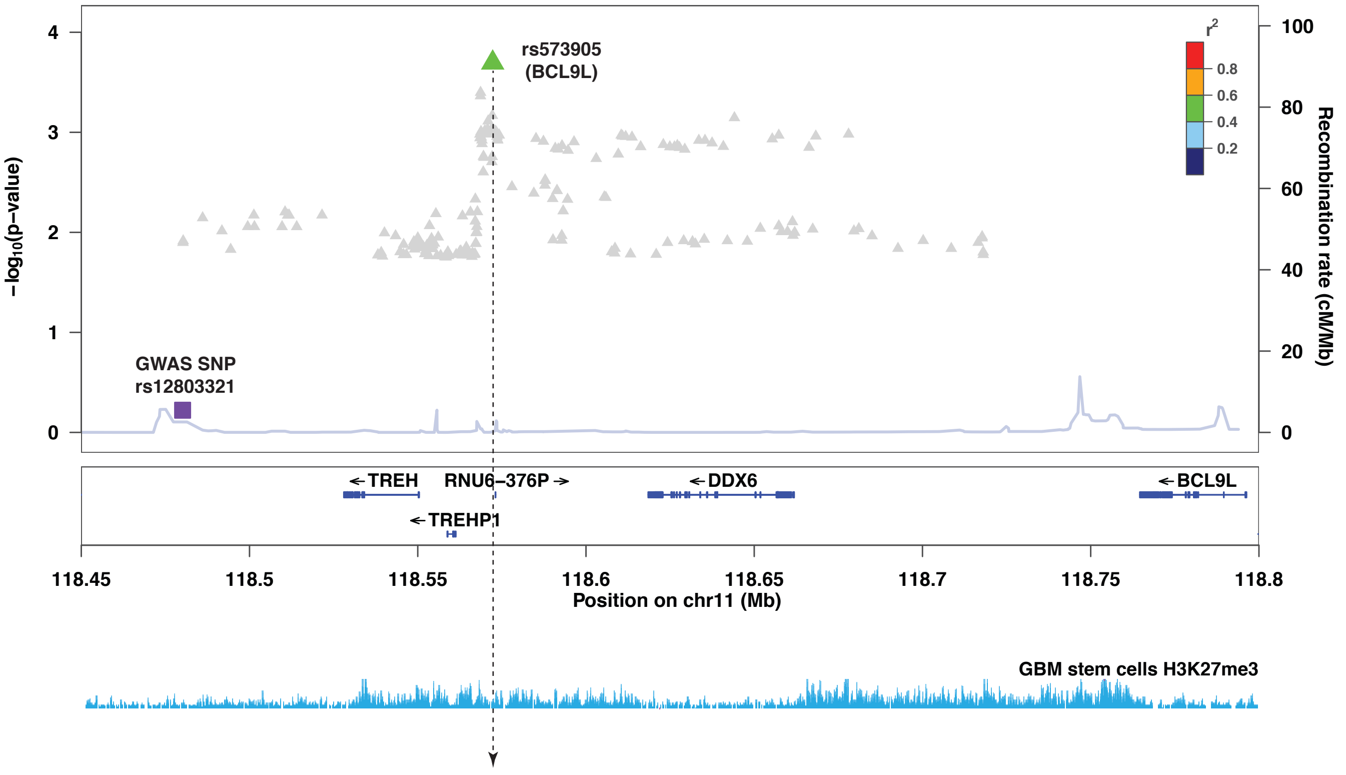

**Supplementary Figure 2G. 15q24.2**

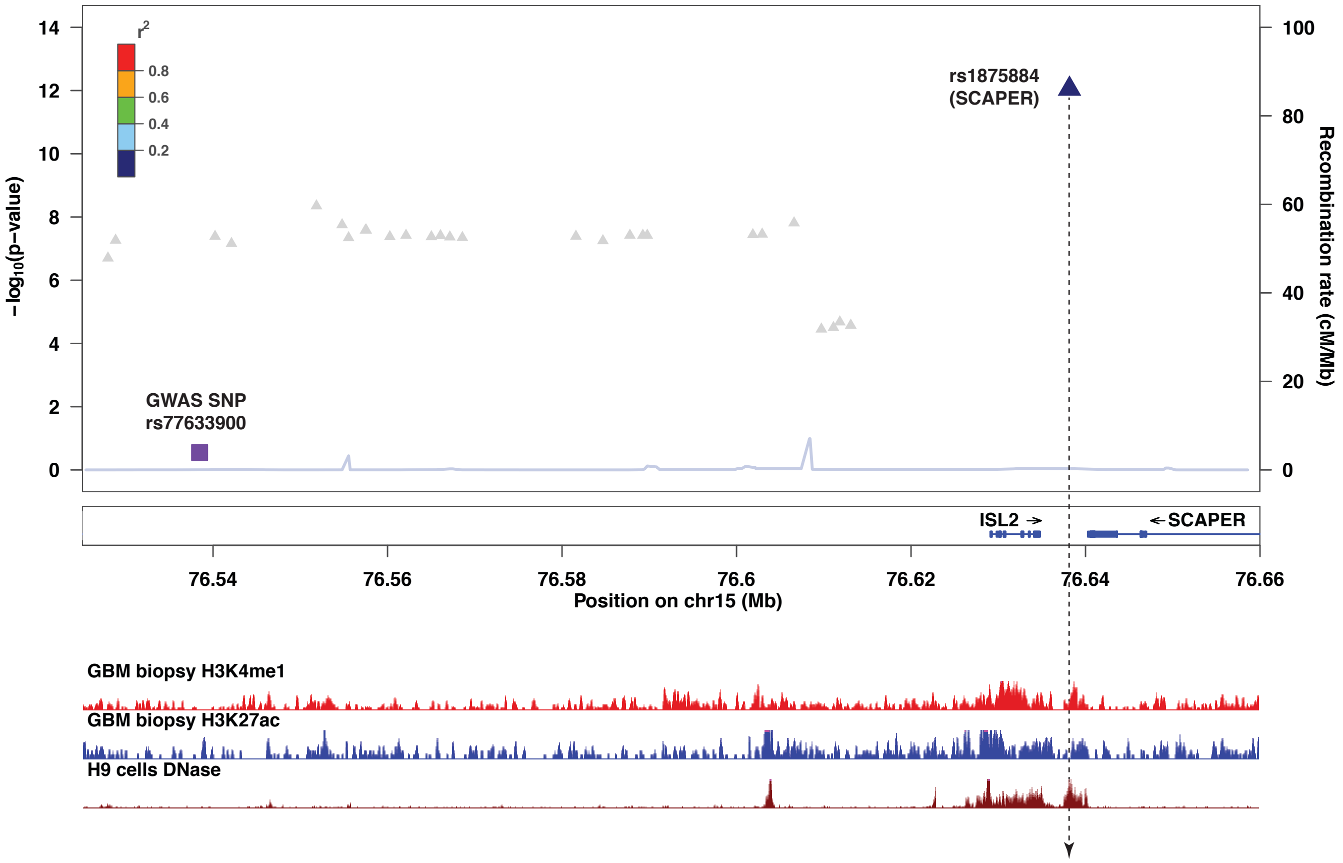

## Supplementary Figure 2H. 16q12.1

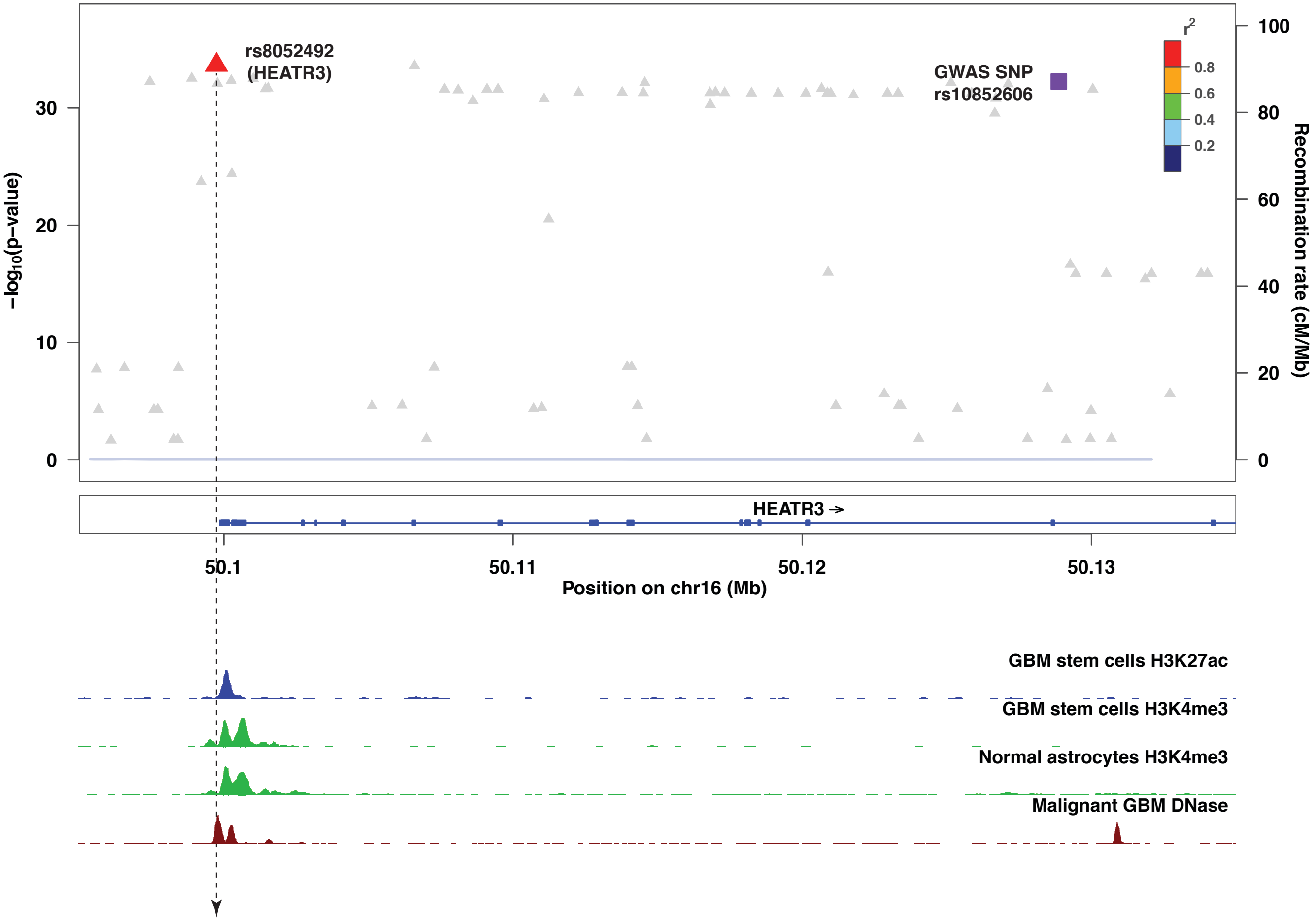

Supplement: Supplementary Figure 1 — Overall study schema. Workflow showing the analyses and results of individual datasets, meta-analysis and colocalization. [file Data_Sheet_1.zip › 609657 Supplementary Material/Figure 2.PDF]
