# Supplementary material for: Meta-Analyses of Splicing and Expression Quantitative Trait Loci Identified Susceptibility Genes of Glioma
Source: Front Genet. 2021 Apr 15;12:609657. doi: 10.3389/fgene.2021.609657 (PMC8081720; doi:10.3389/fgene.2021.609657)

Supplementary Figure 3A. 1q44

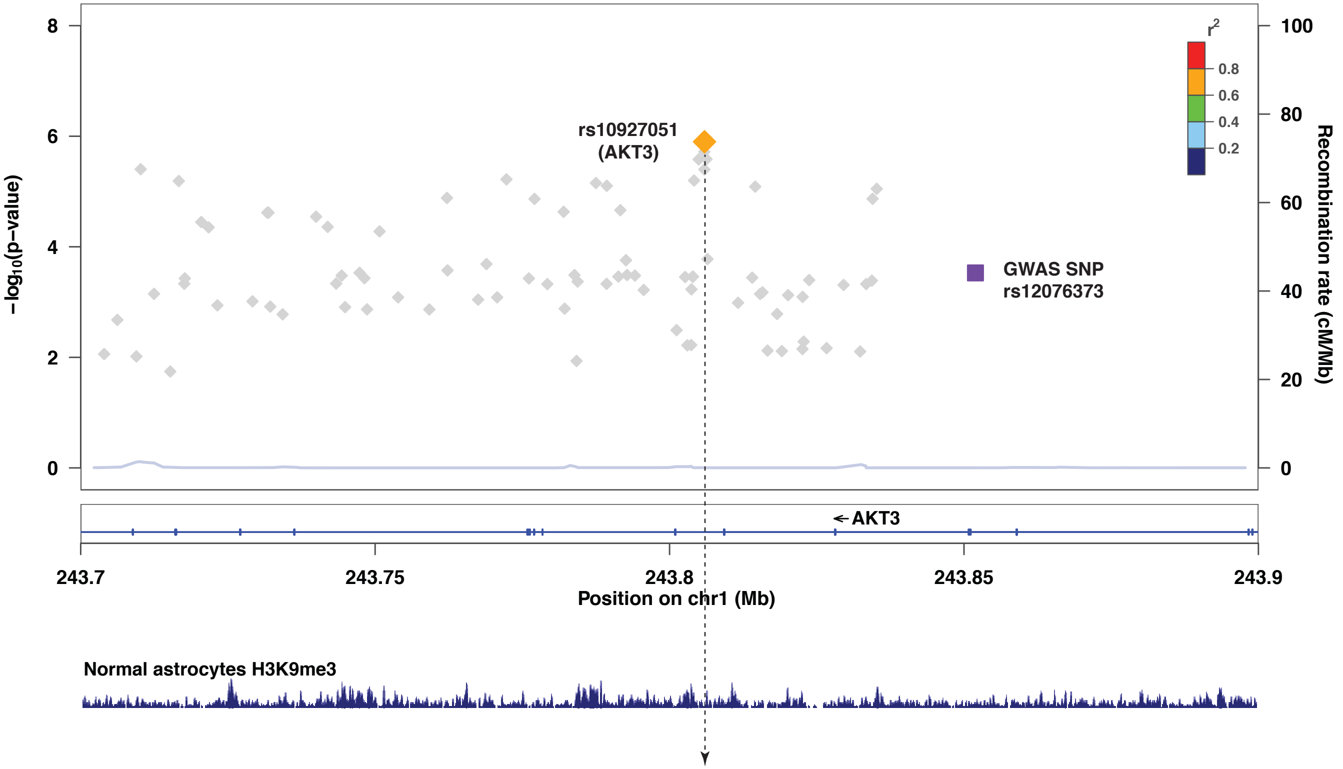

Supplementary Figure 3B. 2q33.3

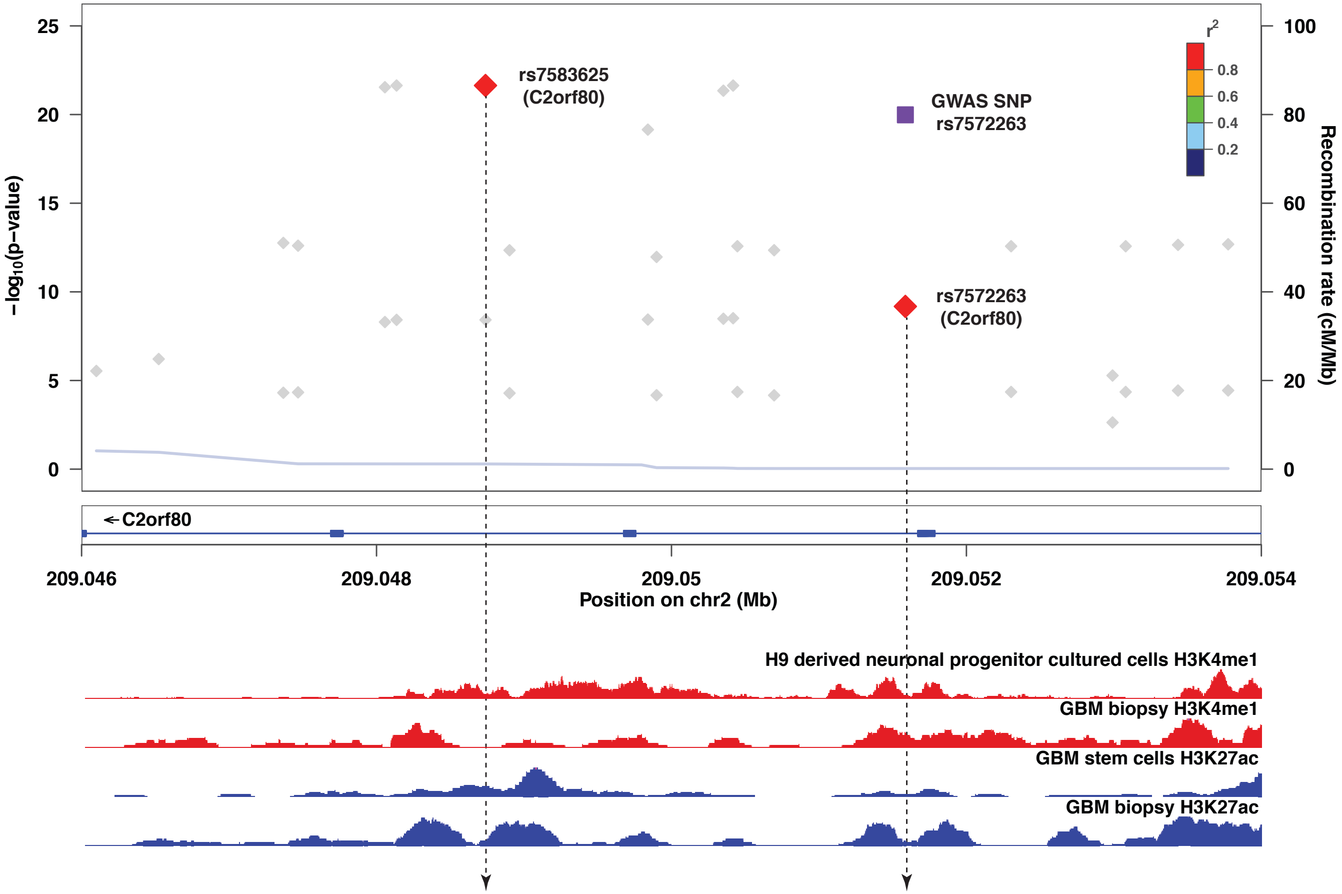

Supplementary Figure 3C. 7p11.2

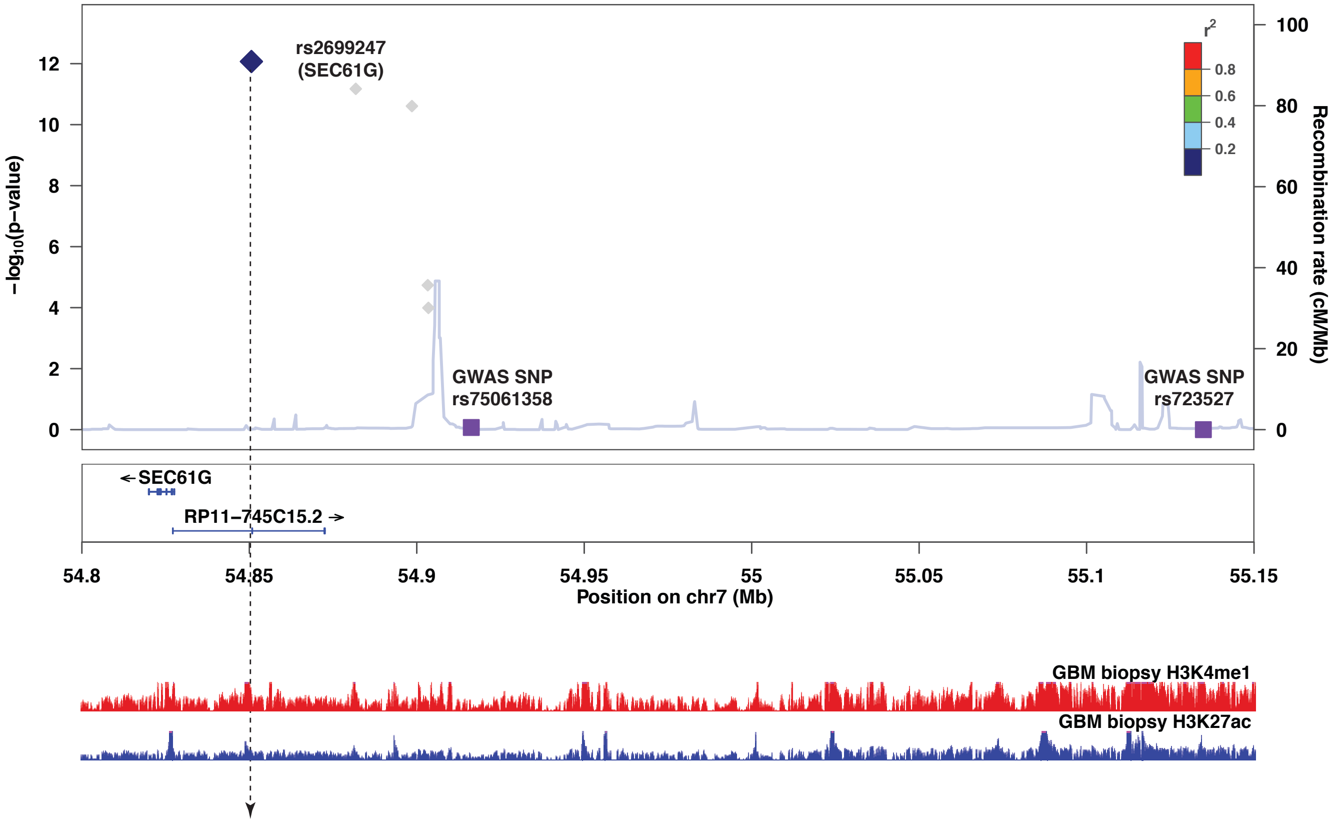

Supplementary Figure 3D. 15q24.2

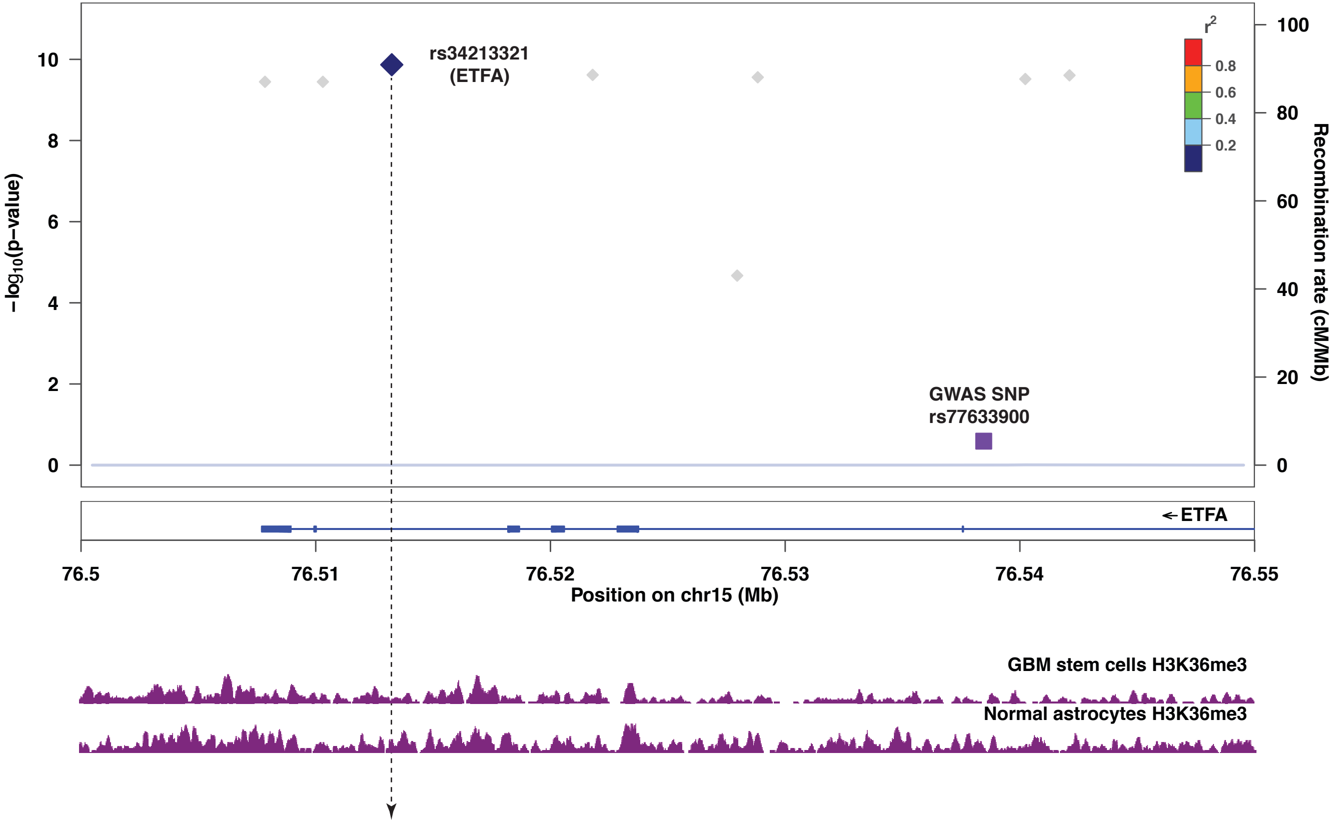

Supplementary Figure 3E. 22q13.1

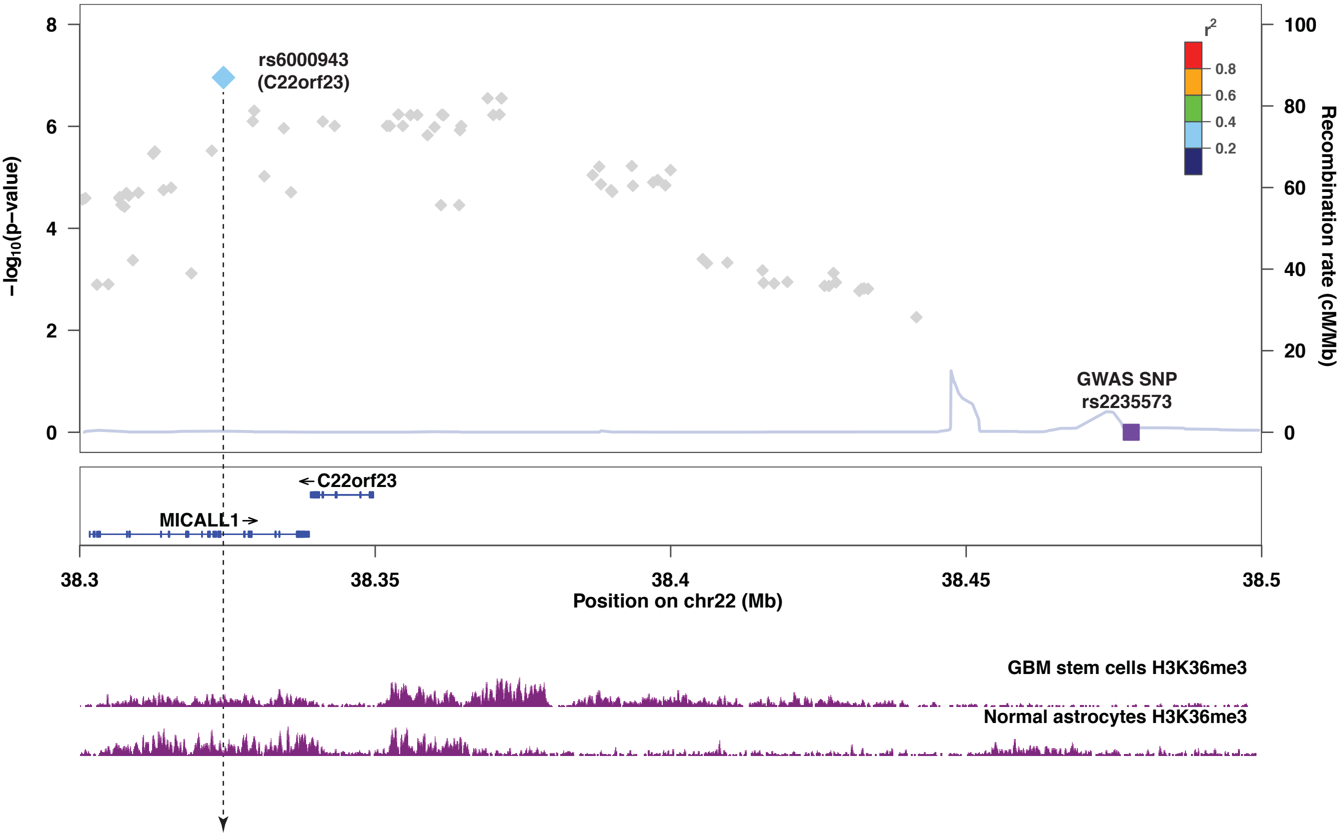

Supplement: Supplementary Figure 1 — Overall study schema. Workflow showing the analyses and results of individual datasets, meta-analysis and colocalization. [file Data_Sheet_1.zip › 609657 Supplementary Material/Figure 3.PDF]
