# Supplementary material for: Meta-Analyses of Splicing and Expression Quantitative Trait Loci Identified Susceptibility Genes of Glioma
Source: Front Genet. 2021 Apr 15;12:609657. doi: 10.3389/fgene.2021.609657 (PMC8081720; doi:10.3389/fgene.2021.609657)
Supplement: Supplementary Figure 1 — Overall study schema. Workflow showing the analyses and results of individual datasets, meta-analysis and colocalization. [file Data_Sheet_1.zip › 609657 Supplementary Material/Table 1.PDF]

Supplementary Table 1. Demographics of individuals included in the analysis

	**CMC release 1 (N = 216)**	**GTEx version 7 (N = 138)**
European Ancestry	100%	100%
Range Age at Death*	17 - 90+	20 - 70
Mean Age (SE) at Death	65.66 (1.31)	57.83 (0.88)
Sex (% Female)	42.59	32.61
Post-mortem interval (PMI) (mean hours (SE))	14.45 (0.53)	GTEx PMI criteria within 24 hrs^
Mean RNA Integrity Number (RIN) score (SE)	7.80 (0.06)	7.15 (0.03)

* Individuals with age >90 have been censored as 90+ for HIPAA compliance in CMC release 1 (https://www.synapse.org/#!Synapse:syn3354385)
^Precise hours not provided by GTEx.
