# Supplementary material for: Meta-Analyses of Splicing and Expression Quantitative Trait Loci Identified Susceptibility Genes of Glioma
Source: Front Genet. 2021 Apr 15;12:609657. doi: 10.3389/fgene.2021.609657 (PMC8081720; doi:10.3389/fgene.2021.609657)
Supplement: Supplementary Figure 1 — Overall study schema. Workflow showing the analyses and results of individual datasets, meta-analysis and colocalization. [file Data_Sheet_1.zip › 609657 Supplementary Material/Table 2.PDF]

Supplementary Table 2: ChIP-seq and cell lines resources. Details of the public datasets used for epigenomic annotations as illustrated in Table 3 and Table 4.

Cell lines	ChIP-seq	Source	Link
GBM stem cells	H3K4me1	NCBI GEO	https://www.ncbi.nlm.nih.gov/geo/query/acc.cgi?acc=GSM1121881
GBM stem cells	H3K4me3	NCBI GEO	https://www.ncbi.nlm.nih.gov/geo/query/acc.cgi?acc=GSM1121882
GBM stem cells	H3K27ac	NCBI GEO	https://www.ncbi.nlm.nih.gov/geo/query/acc.cgi?acc=GSM1121878
GBM stem cells	H3K27me3	NCBI GEO	https://www.ncbi.nlm.nih.gov/geo/query/acc.cgi?acc=GSM1121879
GBM stem cells	H3K36me3	NCBI GEO	https://www.ncbi.nlm.nih.gov/geo/query/acc.cgi?acc=GSM1121880
Primary GBM	H3K27ac	NCBI GEO	https://www.ncbi.nlm.nih.gov/geo/query/acc.cgi?acc=GSM1824800
GBM biopsy	H3K4me1	NCBI GEO	https://www.ncbi.nlm.nih.gov/geo/query/acc.cgi?acc=GSM1866116
GBM biopsy	H3K4me3	NCBI GEO	https://www.ncbi.nlm.nih.gov/geo/query/acc.cgi?acc=GSM1866117
GBM biopsy	H3K27ac	NCBI GEO	https://www.ncbi.nlm.nih.gov/geo/query/acc.cgi?acc=GSM1866064
Normal Astrocytes	H3K4me1	ENCODE	https://www.ncbi.nlm.nih.gov/geo/query/acc.cgi?acc=GSM733710
Normal Astrocytes	H3K4me3	ENCODE	https://www.ncbi.nlm.nih.gov/geo/query/acc.cgi?acc=GSM733747
Normal Astrocytes	H3K27ac	ENCODE	https://www.ncbi.nlm.nih.gov/geo/query/acc.cgi?acc=GSM733763
Normal Astrocytes	H3K9me3	ENCODE	https://www.ncbi.nlm.nih.gov/geo/query/acc.cgi?acc=GSM1003491
Normal Astrocytes	H3K36me3	ENCODE	https://www.ncbi.nlm.nih.gov/geo/query/acc.cgi?acc=GSM733751
Malignant GBM	DNase	ENCODE	https://www.ncbi.nlm.nih.gov/geo/query/acc.cgi?acc=GSM1024773
GBM	DNase	ENCODE	https://www.ncbi.nlm.nih.gov/geo/query/acc.cgi?acc=GSM816668
H9 cells	H3K9me3	Roadmap Epigenomics Project	ftp://ftp.ncbi.nlm.nih.gov/pub/geo/DATA/roadmapepigenomics/by_sample/H9_cell_line/H3K9me3/
H9 cells	DNase	Roadmap Epigenomics Project	ftp://ftp.ncbi.nlm.nih.gov/pub/geo/DATA/roadmapepigenomics/by_sample/H9_cell_line/DNase_hypersensitivity/
H9 derived neuronal progenitor cultured cells	H3K4me1	Roadmap Epigenomics Project	ftp://ftp.ncbi.nlm.nih.gov/pub/geo/DATA/roadmapepigenomics/by_sample/H9_derived_neuronal_progenitor_cultured_cells/H3K4me1/
H9 derived neuronal progenitor cultured cells	H3K9me3	Roadmap Epigenomics Project	ftp://ftp.ncbi.nlm.nih.gov/pub/geo/DATA/roadmapepigenomics/by_sample/H9_derived_neuronal_progenitor_cultured_cells/H3K9me3/
H9 derived neuronal progenitor cultured cells	H3K27me3	Roadmap Epigenomics Project	ftp://ftp.ncbi.nlm.nih.gov/pub/geo/DATA/roadmapepigenomics/by_sample/H9_derived_neuronal_progenitor_cultured_cells/H3K27me3/
