# Supplementary material for: Meta-Analyses of Splicing and Expression Quantitative Trait Loci Identified Susceptibility Genes of Glioma
Source: Front Genet. 2021 Apr 15;12:609657. doi: 10.3389/fgene.2021.609657 (PMC8081720; doi:10.3389/fgene.2021.609657)
Supplement: Supplementary Figure 1 — Overall study schema. Workflow showing the analyses and results of individual datasets, meta-analysis and colocalization. [file Data_Sheet_1.zip › 609657 Supplementary Material/Table 7.PDF]

Supplementary Table 7. eQTL SMR statistics. SMR and HEIDI statistics for the significant colocalized eQTL results reported in Table 1.

Loci	GWAS SNP	Colocalized Regulatory SNP	A1/A2 (Co. Reg SNP)	A1 Freq (Co. Reg SNP)	r^2 (Co. Reg SNP & GWAS SNP)	Target Gene	β (SE) (SMR)	P-value (SMR)	P-value (HEIDI)	N of SNPs in HEIDI test
1p31.3	rs12752552	rs2780816	A/C	0.24	0.45	JAK1	2.73 (0.72)	1.41E-04	1.57E-01	19
2q33.3	rs7572263	rs11883992	A/T	0.18	0.66	C2orf80	-1.73 (0.46)	1.77E-04	8.63E-01	8
5p15.33	rs10069690	rs7712562	G/A	0.84	0.23	TERT	2.73 (0.75)	3.02E-04	2.45E-01	11
7p11.2	rs723527<br>rs75061358	rs80013346	A/G	0.11	0.05<br>0.87	EGFR	-1.73 (0.37)	2.68E-06	3.48E-01	16
9p21.3	rs634537	rs2106120	T/G	0.47	0.62	CDKN2B-AS1	-2.6 (0.51)	3.27E-07	5.03E-01	20
		rs2518723	T/C	0.48	0.53	CDKN2B	-1.93 (0.41)	3.21E-06	7.94E-02	20
10q24.33	rs11598018	rs10883948	T/G	0.54	1	RP11-541N10.3	-1.53 (0.41)	1.74E-04	6.40E-01	6
11q23.3	rs12803321	rs573905	G/A	0.54	0.52	BCL9L	-3.76 (1.09)	5.46E-04	3.13E-01	11
15q24.2	rs77633900	rs1875884	T/C	0.51	0.06	SCAPER	2.09 (0.48)	1.13E-05	8.96E-02	5
16q12.1	rs10852606	rs8052492	G/A	0.29	0.99	HEATR3	1.04 (0.17)	1.73E-09	1.22E-01	20
20q13.33	rs2297440	rs4809318	A/G	0.21	0.29	GMEB2	-4.29 (1.13)	1.57E-04	6.24E-01	6
		rs6062497	T/C	0.71	0.49	ARFRP1	4.29 (1.06)	5.02E-05	5.08E-02	20
		rs909334	A/C	0.21	0.62	STMN3	-3.88 (0.89)	1.33E-05	8.54E-02	20
22q13.1	rs2235573	rs5756894	A/C	0.6	0.59	PICK1	1.54 (0.36)	2.43E-05	6.78E-01	19
		rs6000991	C/T	0.61	0.57	SLC16A8	0.86 (0.2)	2.04E-05	9.32E-02	18
