# Supplementary material for: Meta-Analyses of Splicing and Expression Quantitative Trait Loci Identified Susceptibility Genes of Glioma
Source: Front Genet. 2021 Apr 15;12:609657. doi: 10.3389/fgene.2021.609657 (PMC8081720; doi:10.3389/fgene.2021.609657)
Supplement: Supplementary Figure 1 — Overall study schema. Workflow showing the analyses and results of individual datasets, meta-analysis and colocalization. [file Data_Sheet_1.zip › 609657 Supplementary Material/Table 8.PDF]

Supplementary Table 8. sQTL SMR statistics. SMR and HEIDI statistics for the significant SMR associated sQTL results.

Loci	GWAS SNP	Colocalized Regulatory SNP	A1/A2 (Co. Reg SNP)	A1 Freq (Co. Reg SNP)	r^2 (Co. Reg SNP & GWAS SNP)	Target Gene	Genomic Location (hg19) of Alternatively Spliced Intron Cluster Region from Leafcutter	β (SE) (SMR)	P-value (SMR)	P-value (HEIDI)	N of SNPs in HEIDI test
1q44	rs12076373	rs10927051	C/G	0.2	0.71	AKT3	clu_56564-243727150-243736228	-0.63 (0.16)	1.43E-04	3.35E-01	5
2q33.3	rs7572263	rs7572263	G/A	0.24	1	C2orf80	clu_49792-209047771-209054677	0.72 (0.17)	1.28E-05	6.60E-01	5
		rs7583625	G/A	0.25	0.96	C2orf80	clu_49792-209047771-209048618	0.51 (0.1)	3.34E-07	2.67E-01	8
7p11.2	rs723527<br>rs75061358	rs2699247	A/G	0.36	0.03<br>0.07	SEC61G	clu_32096-54825287-54826851	0.31 (0.07)	6.63E-06	7.08E-02	3
		rs2699247	A/G	0.36	0.03<br>0.07	SEC61G	clu_32096-54825287-54826855	-0.35 (0.08)	1.47E-05	1.04E-01	3
11q23.3	rs12803321	rs11216924	G/C	0.14	0.22	TMEM25	clu_9404-118412790-118416834	-0.63 (0.14)	1.25E-05	9.62E-02	3
		rs73023341	G/A	0.14	0.23	TMEM25	clu_9404-118405070-118419942	-1.05 (0.27)	9.27E-05	2.16E-01	4
		rs61900957	T/C	0.09	0.16	TMEM25	clu_9432-118402586-118402865	0.55 (0.12)	1.96E-06	6.25E-01	3
		rs11217021	C/T	0.25	0.43	DDX6	clu_9452-118627996-118630631	1.26 (0.36)	4.22E-04	6.13E-01	4
		rs7125115	A/G	0.38	0.9	PHLDB1	clu_9440-118478414-118484531	0.53 (0.07)	8.89E-15	1.70E-01	20
		rs7125115	A/G	0.38	0.9	PHLDB1	clu_9440-118481241-118484009	-0.79 (0.11)	2.38E-12	4.84E-01	17
		rs7125115	A/G	0.38	0.9	PHLDB1	clu_9440-118481241-118484531	-0.76 (0.11)	4.31E-12	5.67E-01	16
15q24.2	rs77633900	rs34213321	G/A	0.53	0.08	ETFA	clu_20348-76588078-76603691	-0.41 (0.09)	9.44E-06	3.36E-01	3
16p13.3	rs3751667	rs34316274	G/A	0.23	0.75	LMF1	clu_16201-918097-918944	-0.89 (0.25)	4.47E-04	8.35E-01	4
		rs34316274	G/A	0.23	0.75	RP11-161M6.2	clu_16093-1030705-1031145	-0.51 (0.14)	2.27E-04	3.85E-01	6
		rs34316274	G/A	0.23	0.75	RP11-161M6.2	clu_16094-1026071-1026778	0.49 (0.14)	3.58E-04	5.19E-01	5
		rs4984741	A/G	0.24	0.8	RP11-161M6.2	clu_16094-1026559-1026778	0.66 (0.19)	4.79E-04	4.23E-01	4
16q12.1	rs10852606	rs12932038	C/T	0.71	1	HEATR3	clu_17583-50100180-50100278	-0.5 (0.11)	9.99E-06	7.39E-01	17
		rs2058815	G/T	0.7	0.92	HEATR3	clu_17585-50109622-50109966	-0.83 (0.21)	1.10E-04	6.16E-01	7
		rs2058815	G/T	0.7	0.92	HEATR3	clu_17585-50110000-50112652	-0.96 (0.27)	3.82E-04	6.37E-01	7
		rs2287197	C/T	0.71	0.99	HEATR3	clu_17584-50102778-50103156	-0.58 (0.11)	1.93E-07	2.37E-01	13
		rs2287197	C/T	0.71	0.99	HEATR3	clu_17584-50102778-50109482	-0.52 (0.09)	3.24E-08	2.44E-01	16
		rs2287197	C/T	0.71	0.99	HEATR3	clu_17584-50103200-50104089	-0.62 (0.12)	4.68E-07	5.38E-01	12
		rs8046344	G/C	0.71	1	HEATR3	clu_17584-50102778-50104055	-0.81 (0.19)	3.06E-05	5.99E-01	12
20q13.33	rs2297440	rs1295810	A/G	0.2	0.78	RTEL1-TNFRSF6B	clu_30381-62321563-62321639	-1.63 (0.29)	2.55E-08	1.14E-01	18
		rs2150910	C/T	0.91	0.25	RTEL1-TNFRSF6B	clu_30376-62294908-62297357	-1.06 (0.25)	1.65E-05	2.16E-01	20
		rs3208007	C/T	0.8	0.99	RTEL1-TNFRSF6B	clu_30380-62320485-62320855	0.59 (0.07)	2.93E-17	1.13E-01	20
		rs4809328	T/C	0.71	0.47	RTEL1-TNFRSF6B	clu_30385-62325841-62326419	0.99 (0.27)	2.45E-04	5.41E-02	3
		rs6062487	T/A	0.11	0.03	TNFRSF6B	clu_30388-62328544-62329633	1.04 (0.29)	4.09E-04	1.19E-01	3
		rs6122154	T/C	0.19	0.75	LIME1	clu_30312-62367538-62368886	1.49 (0.41)	2.69E-04	2.88E-01	11
		rs6122154	T/C	0.19	0.75	LIME1	clu_30312-62368064-62368886	-1.08 (0.24)	8.85E-06	6.11E-02	20
22q13.1	rs2235573	rs6000943	C/T	0.37	0.39	C22orf23	clu_28314-38341132-38343288	0.46 (0.13)	4.02E-04	1.37E-01	5
