# Supplementary figures and images for: Meta-Analyses of Splicing and Expression Quantitative Trait Loci Identified Susceptibility Genes of Glioma
Source: Front Genet. 2021 Apr 15;12:609657. doi: 10.3389/fgene.2021.609657 (PMC8081720; doi:10.3389/fgene.2021.609657)

**Supplementary Figure 1.**

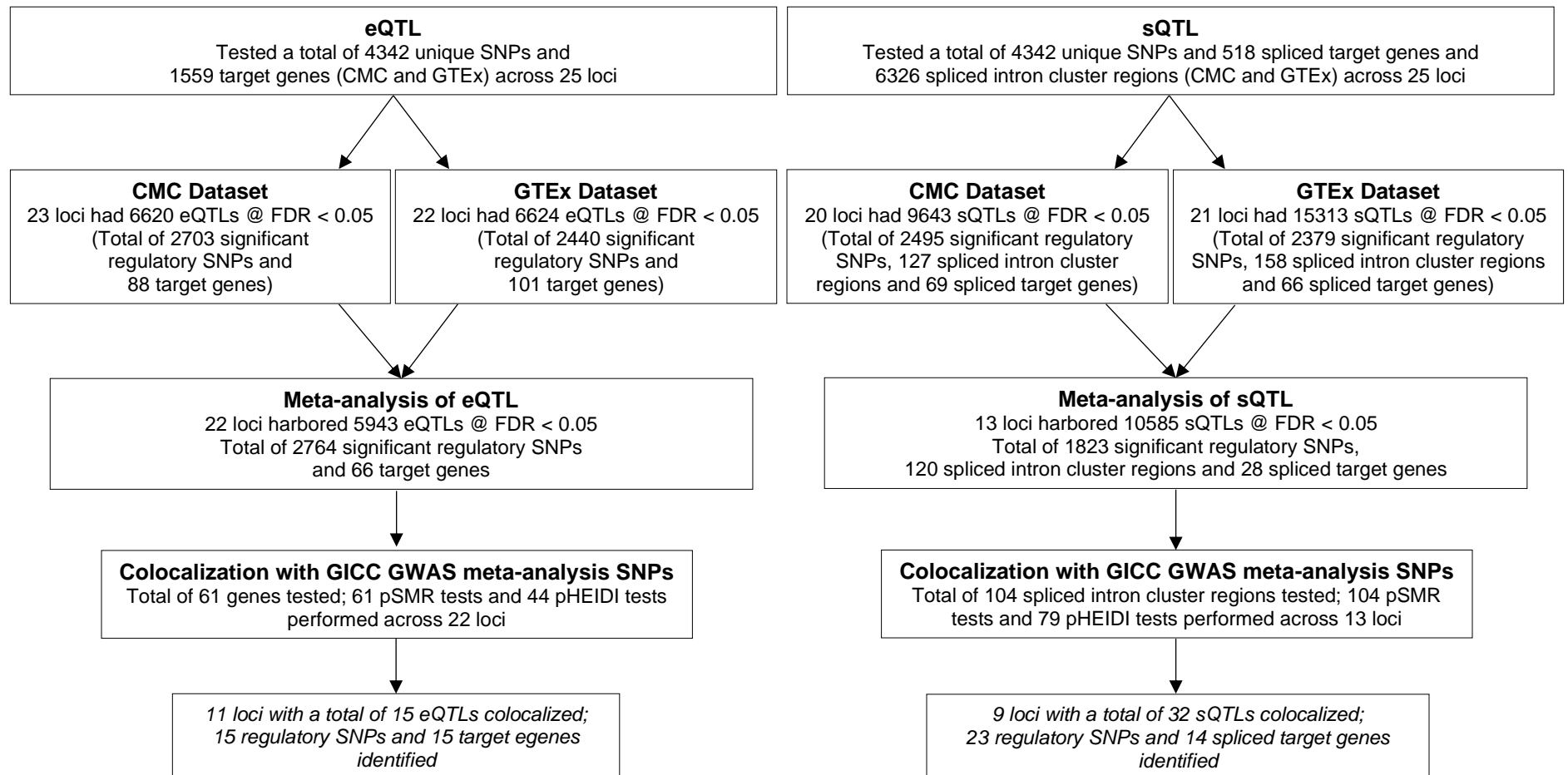

Supplement: Supplementary Figure 1 — Overall study schema. Workflow showing the analyses and results of individual datasets, meta-analysis and colocalization. [file Data_Sheet_1.zip › 609657 Supplementary Material/Figure 1.PDF]

#### 4A. 1q44

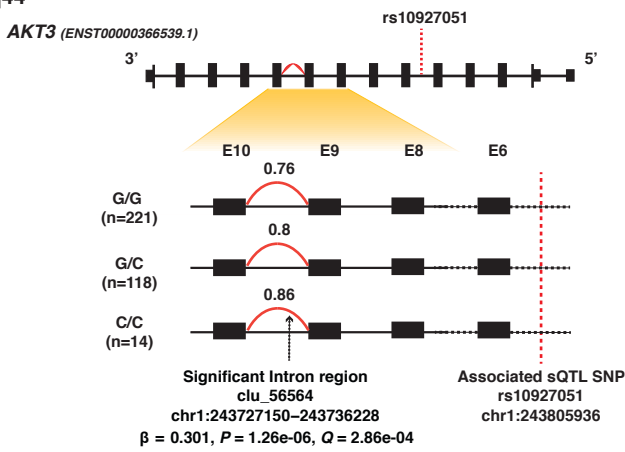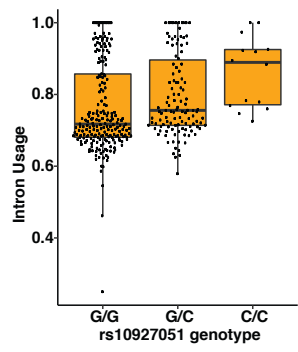

#### 4B. 2q33.3

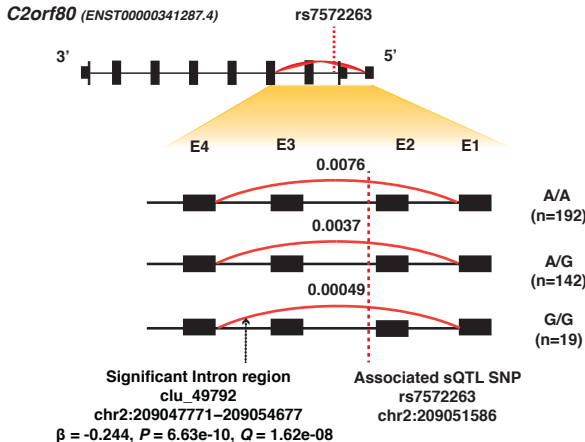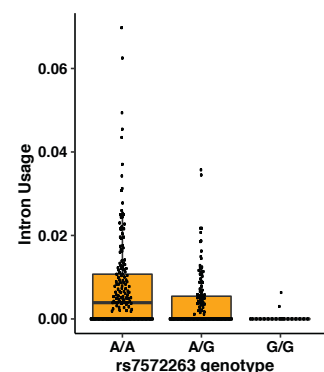

#### 4C. 15q24.2

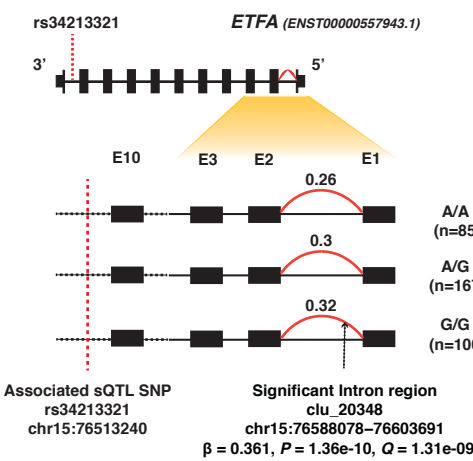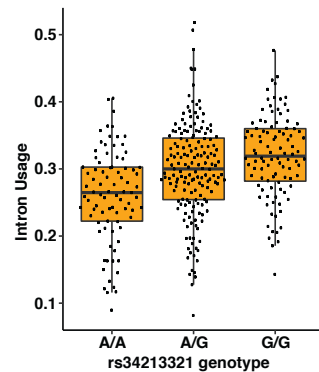

#### 4D. 22q13.1

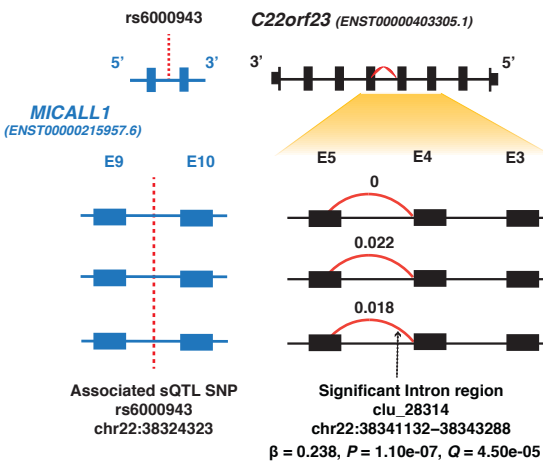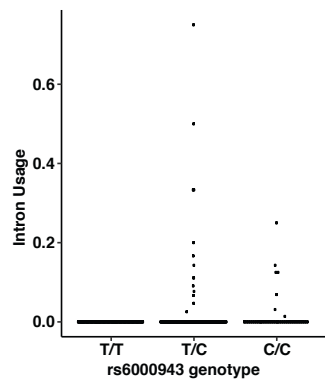

4E. 7p11.2

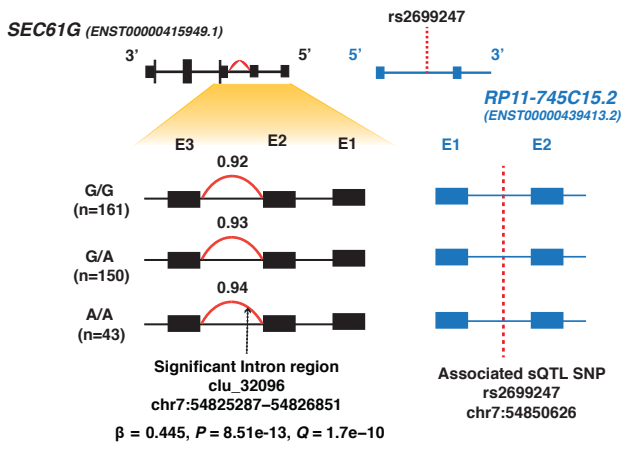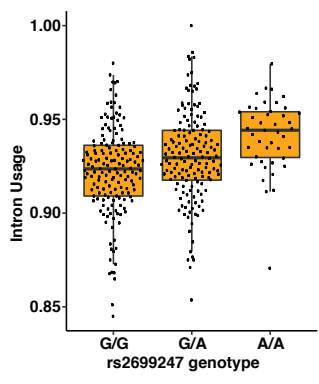

4F. 7p11.2

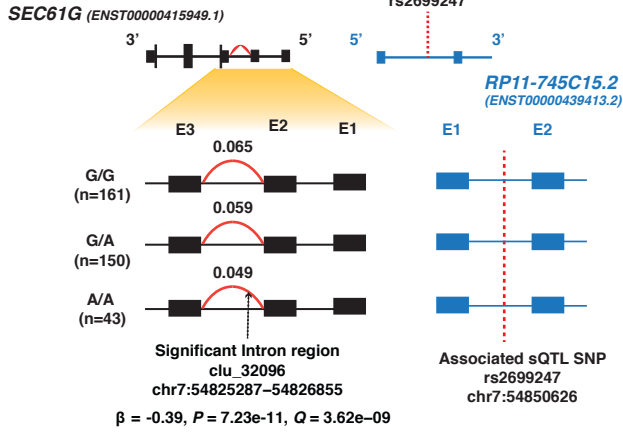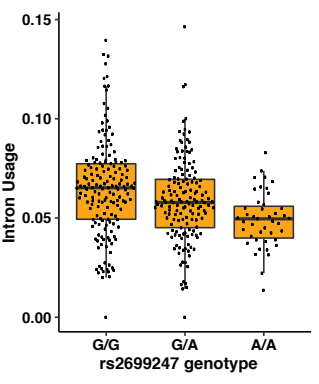

4G. 11q23.3

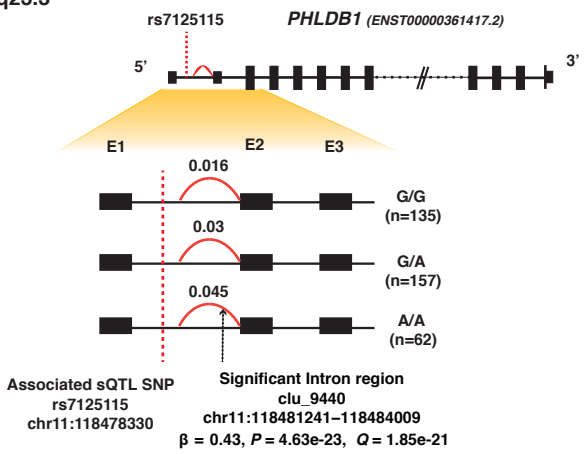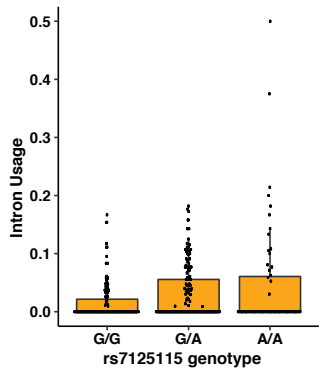

4H. 11q23.3

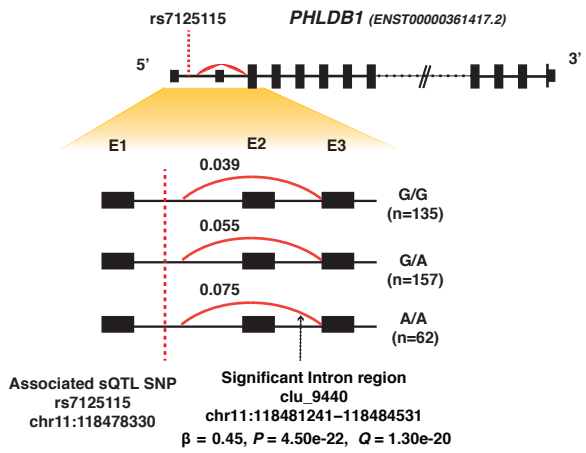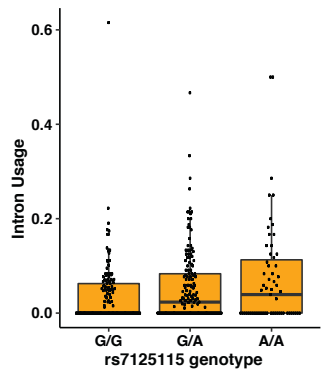

4I. 11q23.3

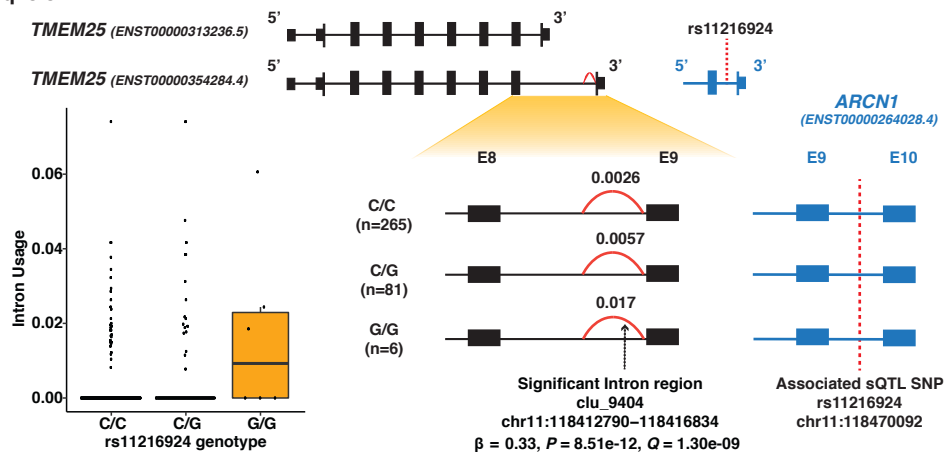

4J. 11q23.3

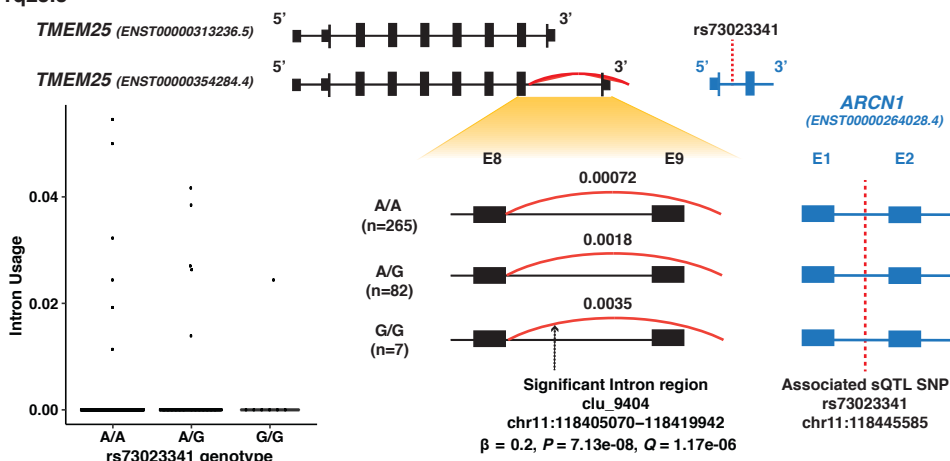

4K. 11q23.3

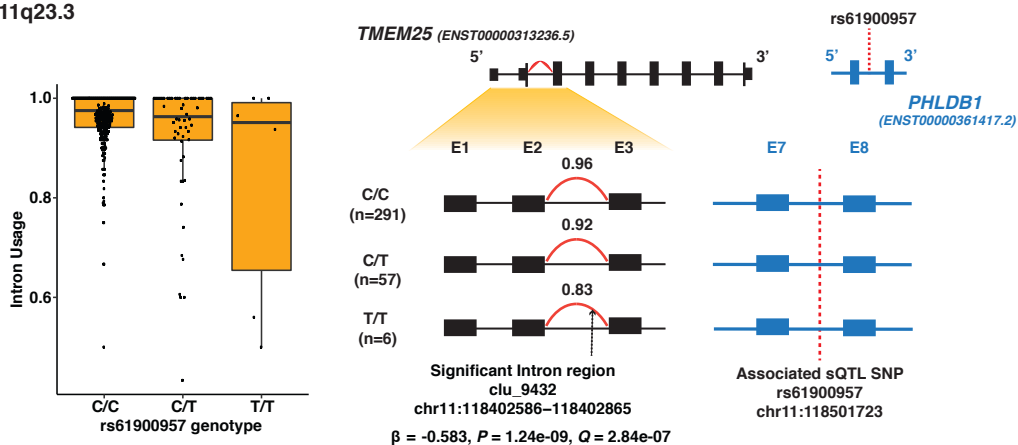

4L. 11q23.3

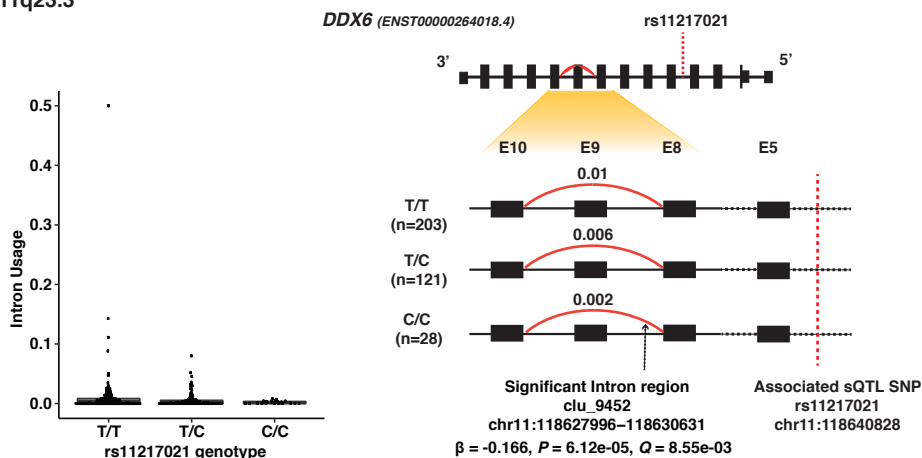

4M. 16p13.3

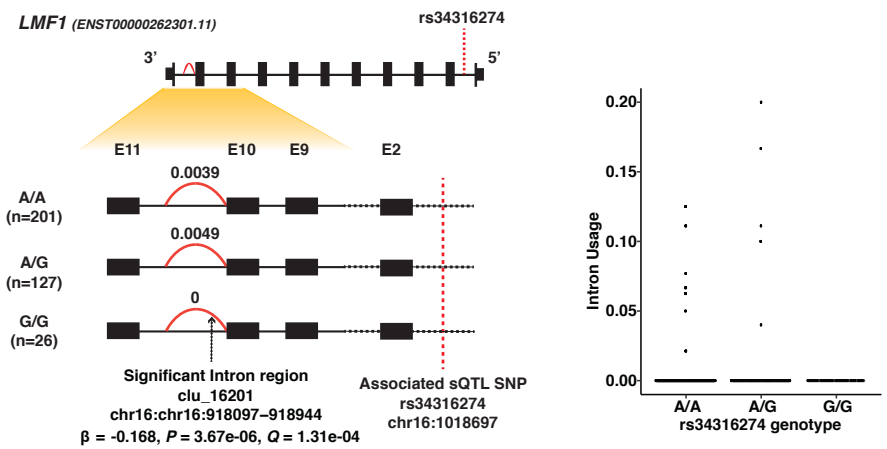

4N. 16p13.3

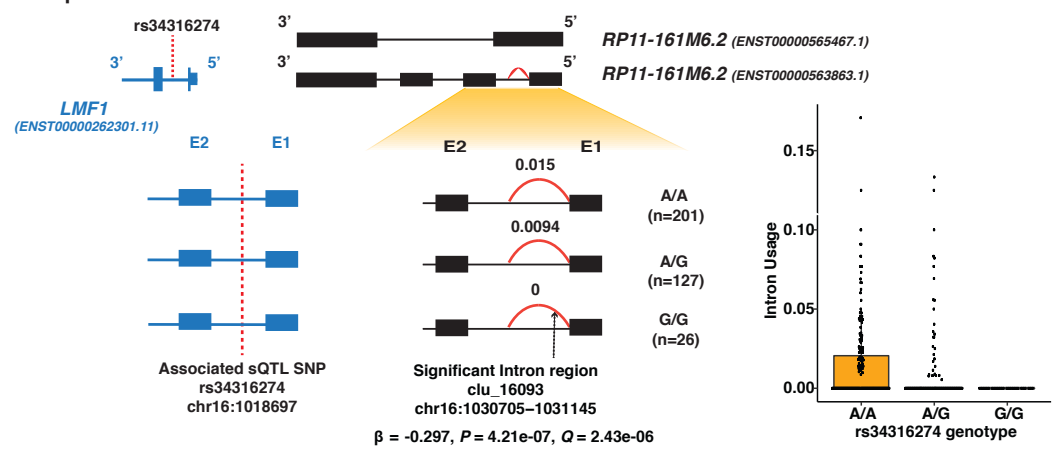

4O. 16p13.3

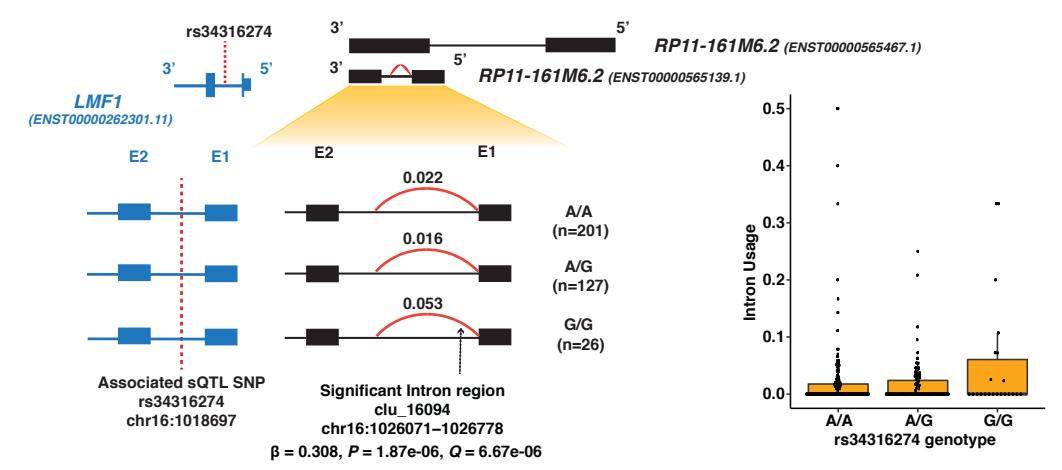

4P. 16p13.3

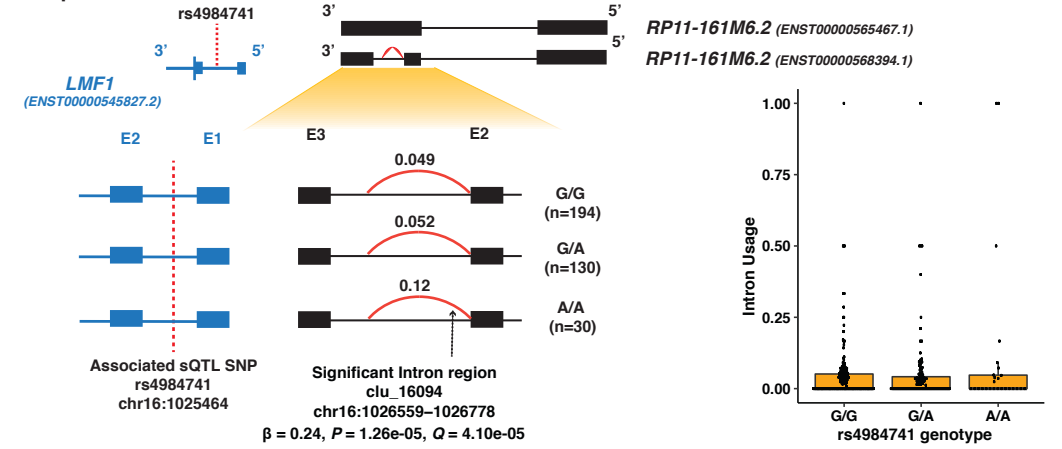

4Q. 16q12.1

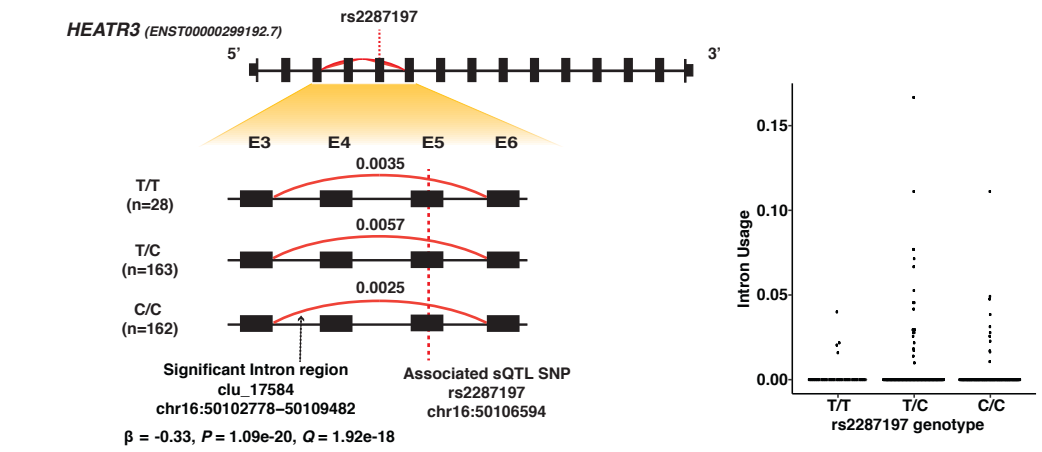

4R. 16q12.1

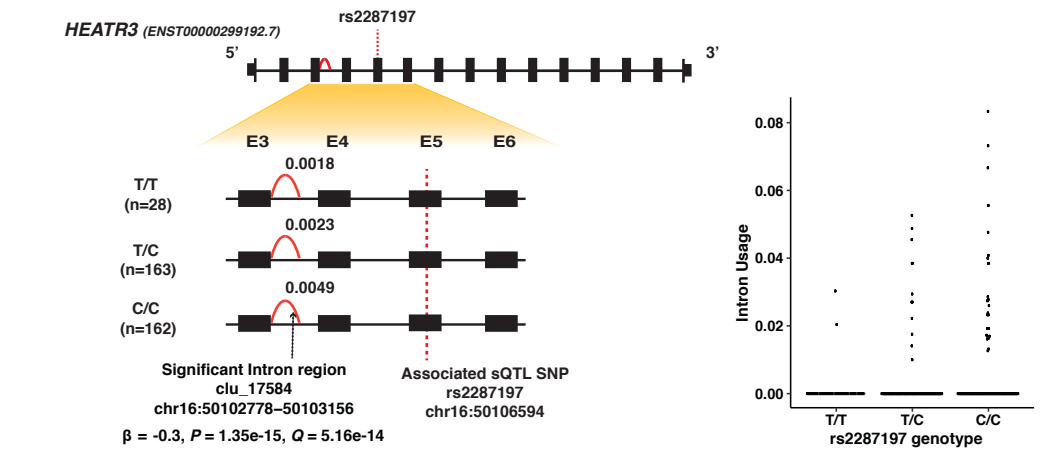

4S. 16q12.1

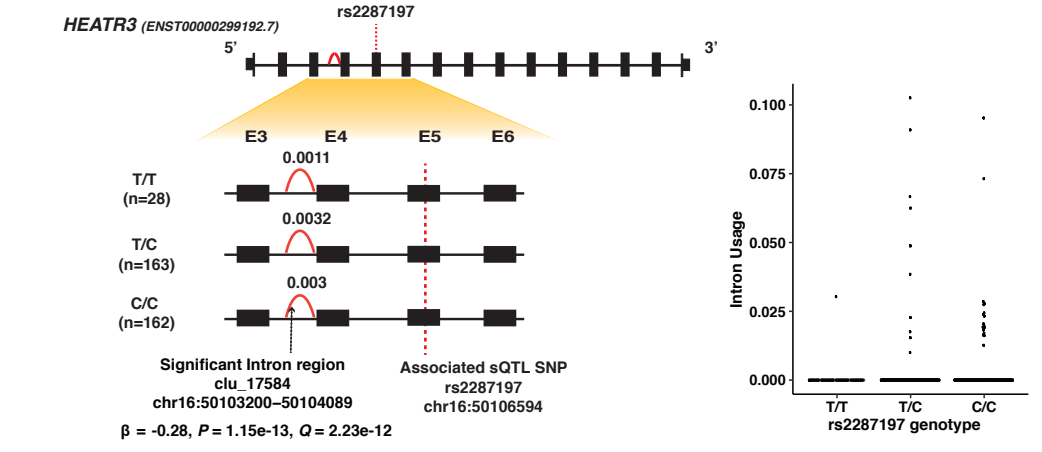

4T. 16q12.1

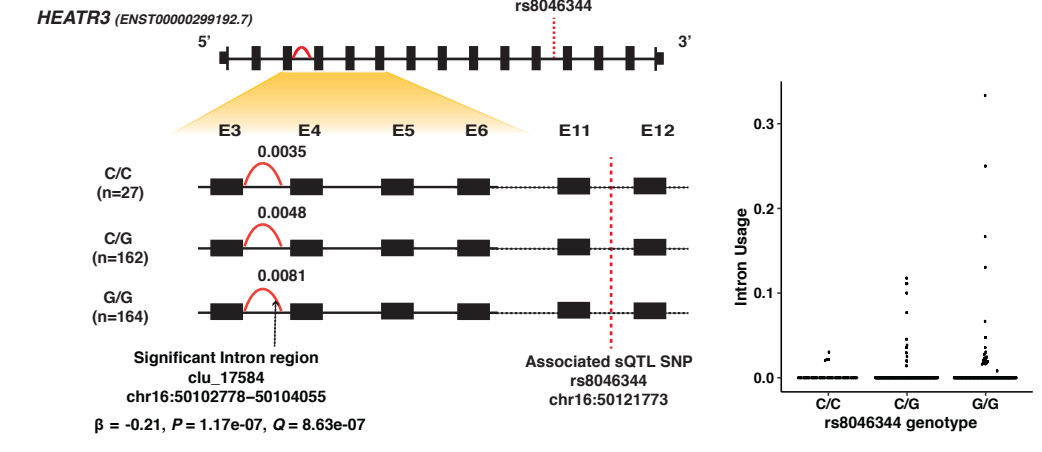

4U. 16q12.1

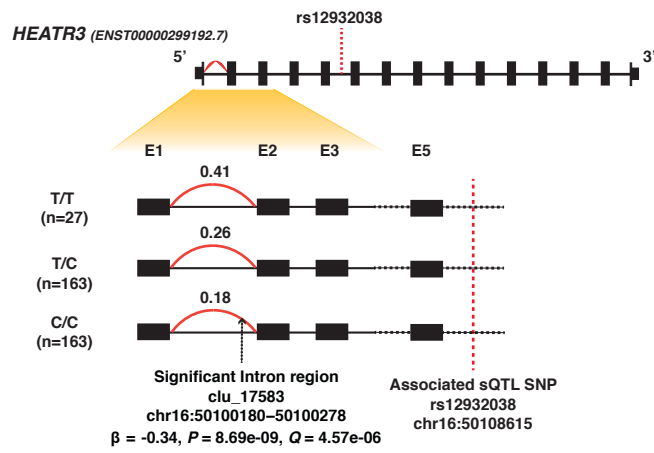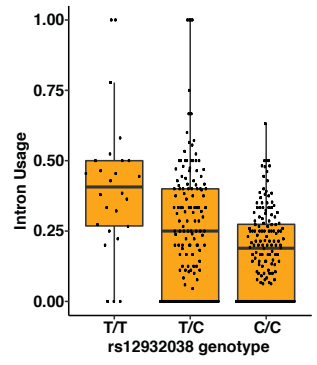

4V. 16q12.1

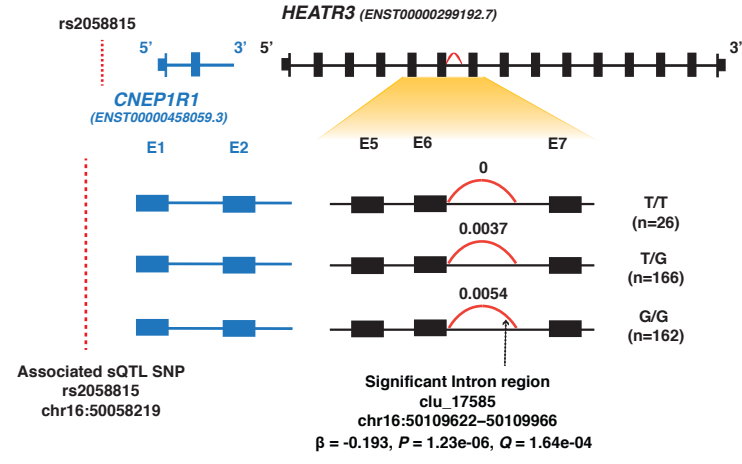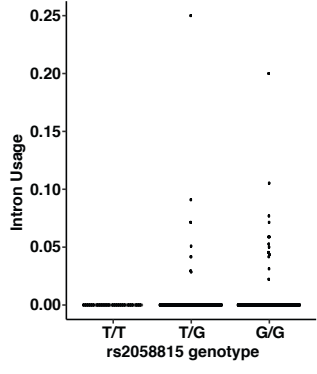

4W. 16q12.1

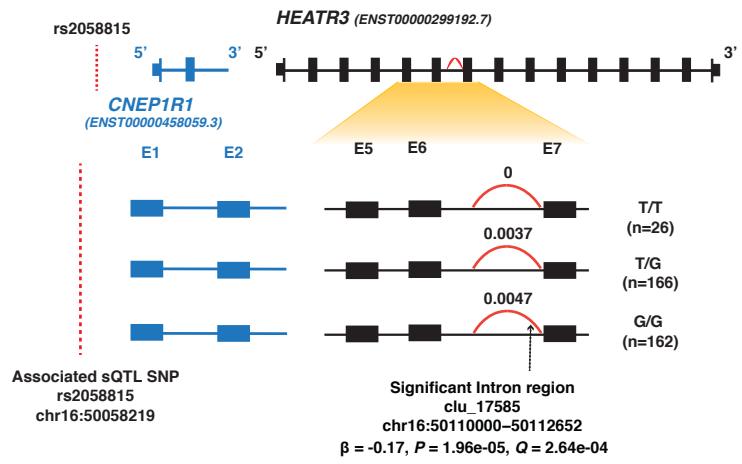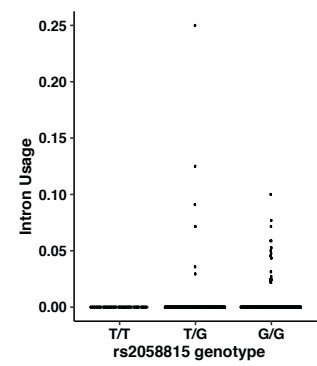

4X. 20q13.33

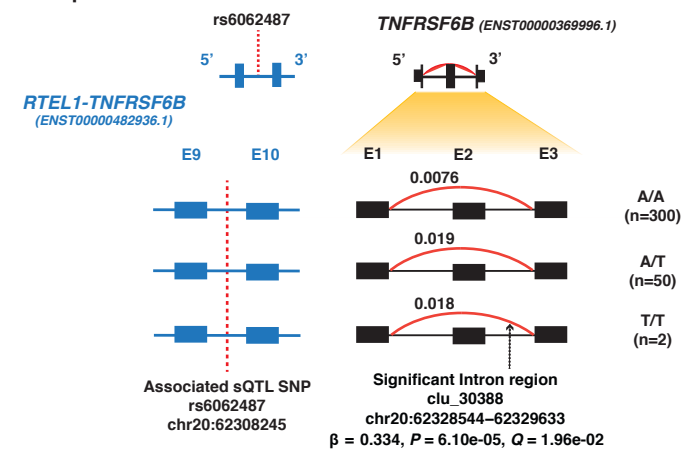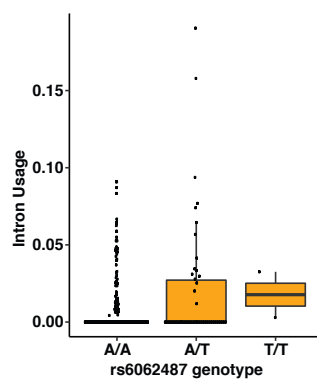

4Y. 20q13.33

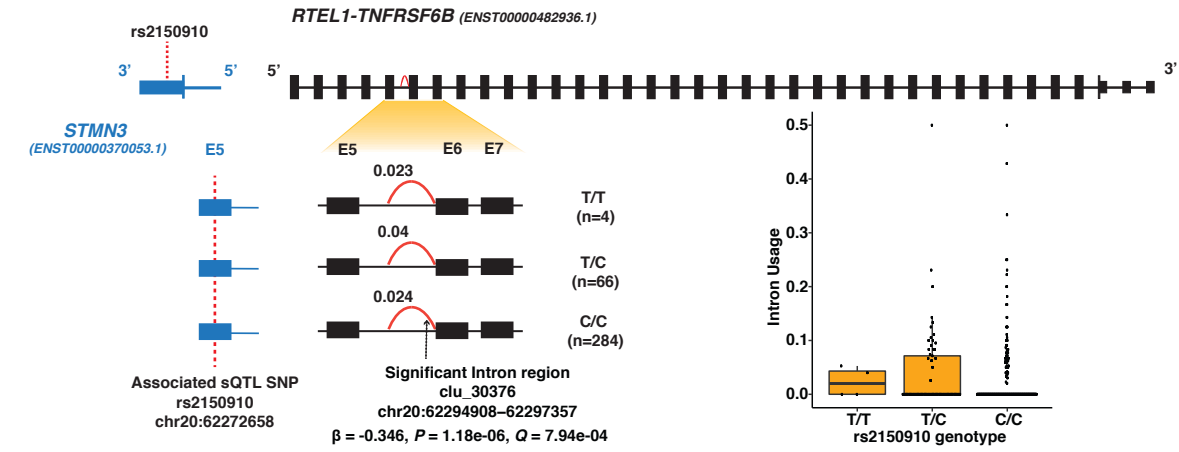

4Z. 20q13.33

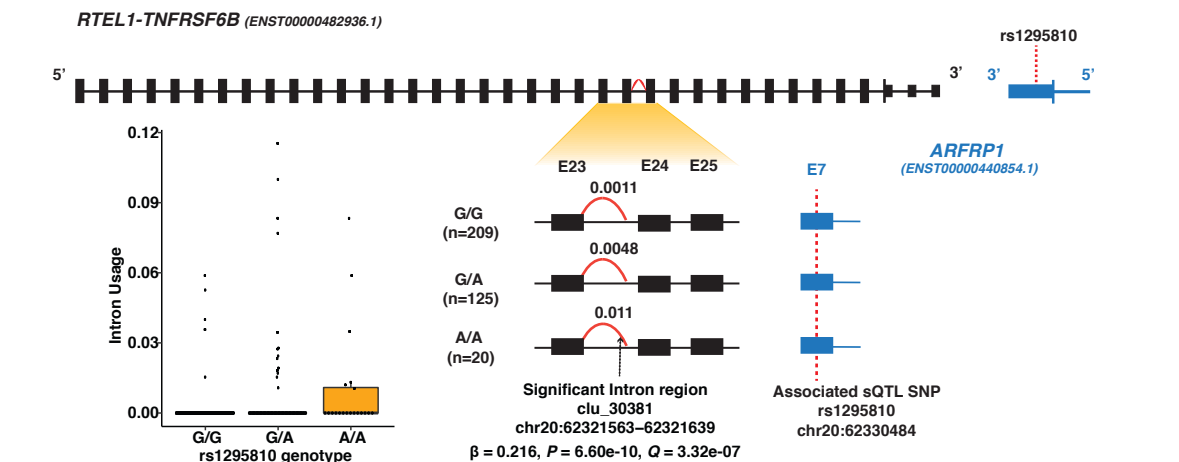

4Aa. 20q13.33

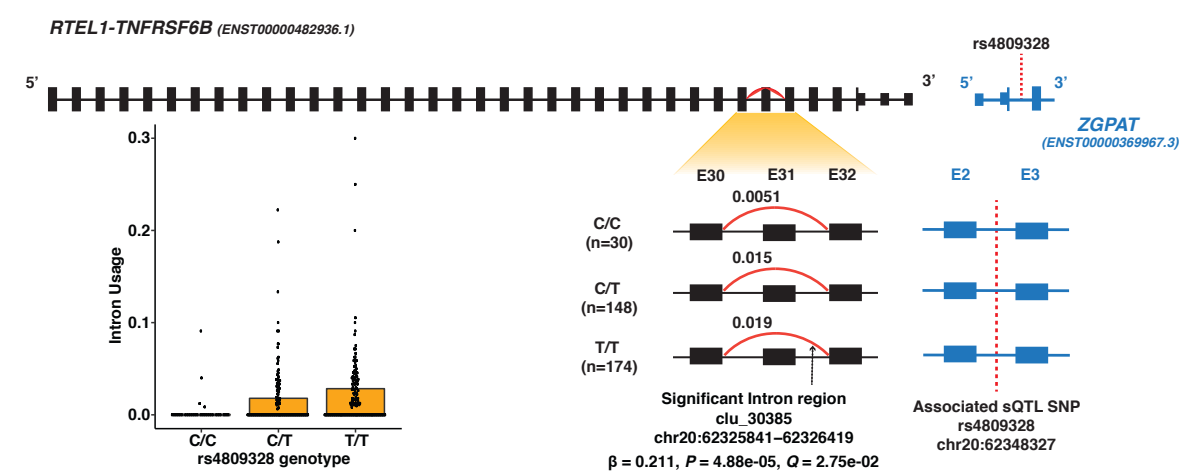

4Ab. 20q13.33

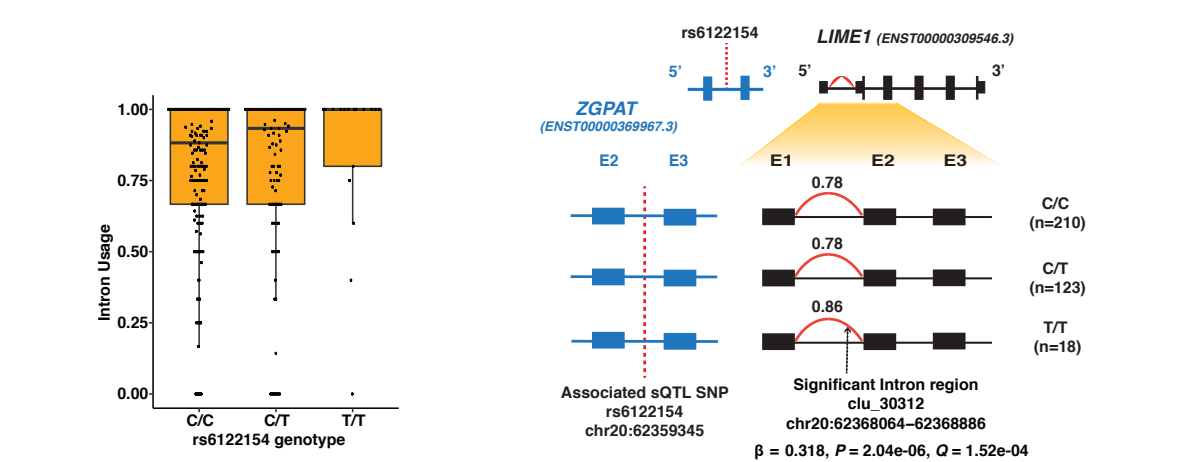

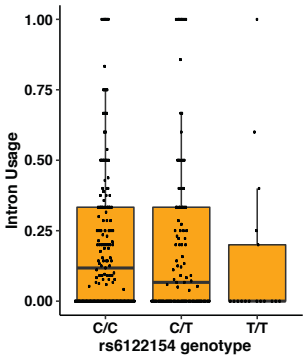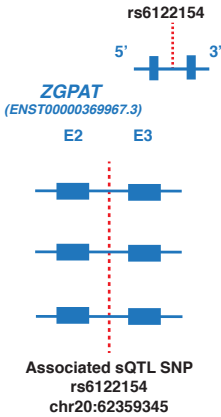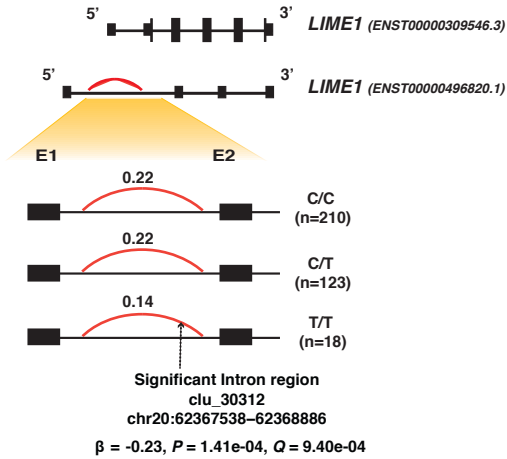

Supplement: Supplementary Figure 1 — Overall study schema. Workflow showing the analyses and results of individual datasets, meta-analysis and colocalization. [file Data_Sheet_1.zip › 609657 Supplementary Material/Figure 4.PDF]
